# A Systematic Review of Multivariate Models for Predicting Fall‐Related Injuries in Older Adults

**DOI:** 10.1155/jonm/1740588

**Published:** 2026-03-24

**Authors:** Yan Cai, Wei Zhu, Xue Zhang, Cong Wang, Shanshan Liu, Yan Jiang

**Affiliations:** ^1^ Center of Gerontology and Geriatrics, West China Hospital, Sichuan University/West China School of Nursing, Sichuan University, Chengdu, Sichuan, China, scu.edu.cn; ^2^ Evidence-Based Nursing Center, West China Hospital, Sichuan University, Chengdu, Sichuan, China, scu.edu.cn; ^3^ Sichuan Provincial Engineering Research Center of Medical Nursing Equipment and Materials, Chengdu, Sichuan, China, scu.edu.cn; ^4^ Department of Neurosurgery, West China Hospital, Sichuan University, Chengdu, Sichuan, China, scu.edu.cn; ^5^ Department of Nursing, West China Hospital, Sichuan University / West China School of Nursing, Sichuan University, Chengdu, Sichuan, China, scu.edu.cn

**Keywords:** fall-related injuries, older adults, prediction model, risk assessment, systematic review

## Abstract

**Aim:**

To evaluate the quality, risk of bias, and clinical applicability of prediction models for fall‐related injuries in older adults.

**Background:**

Numerous prediction models for fall‐related injuries in older adults have been developed, but their quality and applicability in clinical practice and future research remain uncertain.

**Methods:**

We systematically searched Medline (via OVID), Embase (via OVID), Cochrane Library, CINAHL (via EBSCO), Web of Science, and Scopus from inception to May 23, 2024, for English‐language publications. All observational and experimental studies reporting the development or validation of any multivariable prediction model for fall‐related injuries in older adults were included. The risk of bias and applicability was assessed using the PROBAST, and the reporting quality was measured based on the TRIPOD + AI checklist. Data were synthesized using a narrative synthesis approach.

**Results:**

Thirty‐one models from 15 studies were included. Twelve studies focused on the development and/or internal validation of a model, two studies dealt with development and external validation using a nonrandom split‐sample, and one study externally validated existing models. The reported model discriminative statistics exhibited a broad range, from 0.54 to 0.89, in internal or external validation contexts. The risk of applicability was low for all studies, while the overall risk of bias was high in all studies (100.0%). High bias risk was notably prevalent in the analysis domain (100% of studies) and observed in the predictors (33.3%), participants (26.7%), and outcome (6.7%) domains. Median adherence to TRIPOD + AI reporting items was 56.4%.

**Conclusion:**

The discriminative ability in the prediction models of fall‐related injuries in older adults varied widely, with all models exhibiting a high risk of bias according to the PROBAST. Upcoming research should focus on developing high‐quality and reproducible models that undergo proper external validation, followed by studies on implementation.

**Implications for Nursing Management:**

Existing fall‐related injuries prediction models exhibit high bias and inconsistent accuracy, limiting clinical utility. Nursing leaders should advocate for future models that undergo thorough internal and external validation, ensure sufficient events per variable, properly handle missing data, and adopt transparent reporting practices. This will underpin data‐driven clinical decisions and enable targeted fall prevention strategies in vulnerable older adults.

## 1. Introduction

The prevalence of falls among older adults was reported to be 26.5% [1], with 10% of this population experiencing injuries as a result of falls [2], also known as fall‐related injuries (FRI). Annually, approximately 37.3 million falls necessitate medical intervention, and an estimated 684,000 individuals succumb to FRI globally [3]. The development of multivariable prediction models represents a key strategy in precision care for identifying high‐risk individuals to guide targeted prevention [4, 5].

While numerous prediction models for falls exist and have been systematically reviewed [6–8], a critical distinction must be made: risk factors for falling are not identical to those for FRI [9–11]. Consequently, models designed specifically to predict FRI, rather than falls alone, are increasingly important for efficient resource allocation and effective intervention by healthcare workers. Although an increasing number of studies have focused on the development and validation of prediction models for FRI in older adults in recent years, the methodological quality, risk of bias, and clinical applicability of these models remain unassessed in a systematic manner.

To address this gap, this systematic review aims to evaluate the quality, risk of bias, and clinical applicability of prediction models for FRI in older adults that have been developed and published. The findings of this review will offer valuable insights for clinical practice and guide future research in selecting or improving predictive tools. In this review, we employed the PICOTS framework to formulate the research question, as recommended by the guidelines about conducting a systematic review and meta‐analysis of prediction model performance [12, 13]. The principal components of the review question for this systematic review are outlined as follows:

 P (Population): Older adults (people aged 60 years or above).
 I (Index prediction model): Prediction models for FRI that have been developed and published, incorporating at least two predictors.
 C (Comparator): Not applicable.
 O (Outcome): The outcome focused on FRI regardless of severity.
 T (Timing): No restrictions on the chronological order of outcomes.
 S (Setting): Older adults residing in communities, hospitals, or nursing homes.

## 2. Materials and Methods

This systematic review adhered to the Transparent Reporting of Multivariable Prediction Models for Individual Prognosis or Diagnosis for Systematic Reviews and Meta‐Analyses (TRIPOD‐SRMA) guideline [14]. This review was prospectively registered on the International Prospective Register of Systematic Reviews (PROSPERO), under the registration number CRD42024563344.

### 2.1. Search Strategy and Inclusion Criteria

The PICOTS framework was employed to guide the development of the search strategy as well as the inclusion and exclusion criteria for the studies. A comprehensive literature search was conducted across six databases: Medline (via OVID), Embase (via OVID), Cochrane Library, CINAHL (via EBSCO), Web of Science, and Scopus. The search terms and strategy were evaluated by a methodologist with extensive expertise in evidence‐based nursing. Predictive model filters, as proposed and updated by existing studies [15], were incorporated into the search strategy. The retrieval time was from inception to 23 May 2024, restricted to English‐language publications. A search for gray literature was not performed. The search utilized pertinent Medical Subject Headings (MeSH) such as “Accidental Falls,” “Wounds and Injuries,” “Fractures, Bone,” “Logistic Models,” “Algorithms,” “Aged,” and “Aging,” alongside keywords (e.g., “falls,” “predictive model,” “older adults”) and their variations. These terms were strategically combined with the Boolean operators AND and OR. The detailed search strategy is provided in Supporting Information Table 1.

The inclusion criteria for the studies were as follows: (1) Population and setting: The study population was older adults aged ≥ 60 years from any setting (including community‐dwelling, hospitalized, or nursing home residents). (2) Index prediction model: The study reported on the development and/or validation of a prediction model with at least two predictors. (3) Outcome: The outcome of interest was FRI, defined as any physical harm resulting from a fall, regardless of severity (including, but not limited to, bruises, abrasions, sprains, fractures, or head trauma requiring any form of assessment or care). (4) Timing and study design: The study employed an observational (prospective or retrospective) or experimental design. There were no restrictions on the time between predictor assessment and outcome occurrence. (5) When two studies used data from the same cohort but from different years and samples, both were included. Studies were excluded if they met any of the following criteria: (1) addressing topics outside the scope of the review; (2) mixed‐age populations without extractable data for the ≥ 60‐year‐old subgroup; (3) without full‐text availability; and (4) classified as reports, guidelines, letters, editorials, protocols, reviews, or conference abstracts.

### 2.2. Study Selection and Screening

Prior to the screening process, all the search results were imported and consolidated via EndNote X9. Following the automated removal of duplicate entries, two independent researchers evaluated the titles and abstracts of the remaining studies according to the predefined inclusion and exclusion criteria. Discrepancies were addressed through discussion, and unresolved conflicts were referred to a third researcher for further resolution. Studies meeting the inclusion criteria underwent a full‐text screening, with the same methodology employed as that used for the title and abstract screening.

### 2.3. Risk of Bias and Quality Assessment

The Prediction model Risk of Bias Assessment Tool (PROBAST) [16] was applied to evaluate the risk of bias and the applicability of the included studies. The PROBAST checklist serves as a tool for the critical evaluation of studies engaged in the development, validation, or updating of prediction models for individualized predictions. This evaluation encompasses an assessment of participants, predictors, outcomes, and analytical methods to determine the risk of bias, culminating in an overall risk of bias rating. The overall assessment can be categorized as ‘high,’ ‘low,’ or ‘unclear.’ The presence of bias and applicability concerns in the studies was independently assessed by two researchers, and any discrepancies were settled by a third researcher.

Furthermore, the included studies were evaluated for quality and completeness of reporting in accordance with the TRIPOD + AI guideline [17], which represents the updated guidance for the TRIPOD, initially released in 2015 [18]. TRIPOD + AI offers standardized guidance for reporting prediction model studies, regardless of whether regression modeling or machine learning methodologies are employed. A total of 52 subitems were used to evaluate the study’s adherence to the checklist, with each subitem receiving a score of one. The adherence score was determined by dividing the total number of adhered TRIPOD + AI subitems by the number applicable to the specific study. Similarly, the adherence of study abstracts was assessed using the 13‐item TRIPOD + AI for Abstracts checklist.

### 2.4. Data Extraction

A structured data extraction form was created in Microsoft Excel, drawing upon the Checklist for critical appraisal and data extraction for systematic reviews of prediction modelling studies (CHARMS) checklist [19]. This calibrated form was then piloted on five randomly selected included studies, followed by a discussion to resolve discrepancies and align their understandings of the extraction criteria. After piloting, the reviewers compared results and refined the form and instructions to improve clarity. For the main extraction, data from all included studies were extracted independently by the two researchers. Discrepancies were resolved through consensus or by consulting a third senior researcher. Extracted information was categorized into two groups: (1) Basic study and participant characteristics: author, publication year, country, study year, study design, setting, participant age, gender distribution, sample size, outcome, prediction time horizon of outcome, and the number of predictors. (2) Model development, validation, and performance information: the types of prediction model studies, variable selection method, model development or validation techniques, discrimination performance and calibration measures, and the form of model presentation. For the types of prediction model studies, the prediction model studies were categorized according to the TRIPOD guidelines [20] into the following types: type 1a (development only), type 1b (development and validation using resampling), type 2a (random split sample development and validation), type 2b (nonrandom split sample development and validation), type 3 (development and validation using separate data), and type 4 (validation only). The latter three types were regarded as external validation studies.

### 2.5. Data Synthesis

Established methodological guidance indicates that a meta‐analysis of a prediction model’s performance is only recommended when more than five external validation studies are available for the same model to ensure reliable and generalizable pooled estimates [21]. In this review, no single FRI prediction model was externally validated in more than five studies; therefore, a meta‐analysis was not performed. Given the nature of the included evidence, a narrative synthesis was performed. This synthesis followed a three‐step process to ensure a comprehensive and critical integration of findings: (1) Descriptive summary of study characteristics: The included studies were systematically categorized and compared based on key design, population, and outcome features. (2) Descriptive summary of model characteristics: The predictors, methods and results of model development and validation were summarized. (3) Critical appraisal of methodological and reporting quality: This step involved presenting the overall ratings and common methodological shortcomings assessed by the PROBAST tool and the TRIPOD + AI checklist.

## 3. Results

### 3.1. Search Results of Included Studies

**FIGURE 1:**
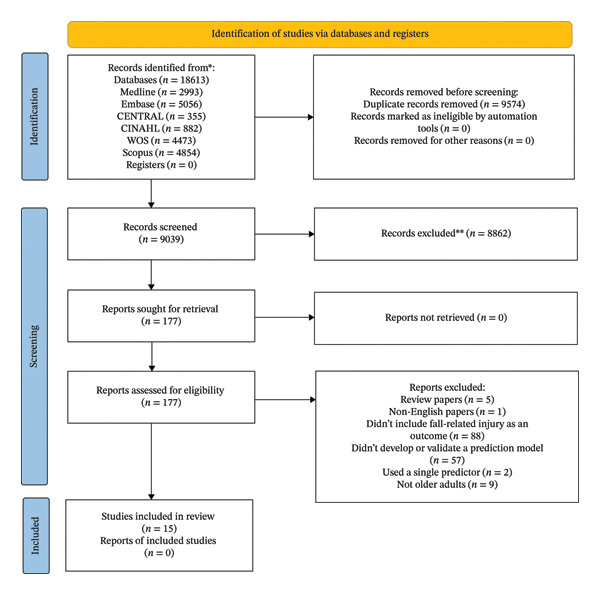
Flow diagram of studies selecting.

A thorough search across six databases resulted in the identification of 18,613 records. Following the removal of 9574 duplicates, 8862 records were excluded based on their titles and abstracts, and 162 records were eliminated after full‐text screening. A list of the excluded studies with the reasons for exclusion was provided in Supporting Information Table 2. This systematic review concluded with the inclusion of 15 studies, and the screening process is depicted in Figure 1.

### 3.2. Characteristics of the Included Studies

Table 1 outlines the overview and characteristics of the studies included. Of these, eight were prospective cohort studies [22–24, 26, 28, 29, 31, 34], three were retrospective cohort studies [25, 27, 30], three were designed as case‐control studies [33, 35, 36], and one was cross‐sectional [32]. The studies included seven conducted in community [22, 23, 26, 28, 29, 31, 34], six in hospitals [24, 27, 30, 33, 35, 36], and two in nursing homes [25, 32]. Among 11 studies, the average or median age of participants was 70 years or older, and the participants in the other four studies were aged 80 years or above [24, 25, 27, 32]. Eight studies had a participant composition of over 60% females [24–27, 29, 32, 34, 35]. The percentage of FRI events ranged from 1.8% to 59.3%. The total sample sizes spanned from 108 to 1,073,334 individuals across the studies.

**TABLE 1:** Overview and characteristics of the included studies.

Author (publication year)	Country (study year)	Study design	Setting	Age of participants (years old)	Proportion of gender (%)	FRI cases (proportion)/analytical sample size	Outcome	Prediction time horizon of outcome
Chan et al. [22]	UK (2013–2022)	Prospective cohort	Community	Mean ± SD: 69.0 ± 3.0	Female (49.5), Male (50.5)	1627 (5.1%)/32,169	FRI	9 years
Chen et al. [23]	China (2015–2018)	Prospective cohort	Community	Mean ± SD: 67.7 ± 6.2	Female (51.3), Male (48.7)	589 (10.1%)/5818	FRI	3 years
Davis et al. [24]	Canada (2010–2015)	Prospective cohort	Hospital	Mean ± SD: 81.5 ± 6.5	Female (69.1), Male (30.9)	64 (59.3%)/108	FRI	1 year
Duprey et al. [25]	USA (2016–2017)	Retrospective cohort	Nursing home	Median (Q1, Q3): 85.0 (77.5, 90.6)	Female (69.6), Male (30.4)	6 months: 25,805 (3.5%)/733,427; 2 years: 43,976 (6.0%)/733,427	FRI	6 months, 2 years
Ek et al. [26]	Sweden (2001–2009)	Prospective cohort	Community	Mean ± SD: 73.0 ± 10.3	Female (62.3), Male (37.7)	392 (14.0%)/2808	First FRI	5 years
Engelbart et al. [27]	USA (2012–2017)	Retrospective cohort	Hospital	Mean ± SD: 81.3 ± 8.4 (Training data), 80.9 ± 8.4 (Testing data)	Female (61.1), Male (38.9)	253 (10.9%)/2312	Fall‐related cervical spine injuries	Diagnostic prediction
Frisendahl et al. [28]	Sweden (2001–2015)	Prospective cohort	Community	Mean ± SD: 71.0 ± 9.6 (Female), 70.0 ± 9.2 (Male) (Cohort 1); 75.0 ± 7.8 (Female), 74.0 ± 8.0 (Male) (Cohort 2)	Female (53.0), Male (47.0) (Cohort 1); Female (54.5), Male (45.5) (Cohort 2)	Cohort 1: The injurious fall rate per 1000 person‐years of 18.7 (95% CI 16.4–21.3)/2272; Cohort 2: The injurious fall rate per 1000 person‐years of 19.7 (95% CI 17.4–22.2)/494	First FRI	5 years
Frisendahl et al. [29]	Sweden (2010–2012)	Prospective cohort	Community	Mean ± SD: 73.6 ± 13.1 (Female), 70.6 ± 12.2 (Male)	Female (62.0), Male (38.0)	Female: The injurious fall rate per 1000 person‐years of 54.9 (95% CI: 47.2–63.8)/740; Male: The injurious fall rate per 1000 person‐years of 36.3 (95% CI: 28.8–45.8)/454	First FRI	5 years
Heo et al. [30]	Korea (2018–2019)	Retrospective cohort	Hospital	Age group proportion: 65–69 (30.6%), 70–74 (25.5%), ≥ 75 (43.9%)	Female (58.5), Male (41.5)	18,791 (1.8%)/1,073,334	FRI	The observation period ends when a specific event occurs (FRI, death or study end)
Li et al. [31]	China (2011–2018)	Prospective cohort	Community	Median (IQR): 65.0–66.0 (8.0–9.0) (Five dataset)	Female (48.6–50.7), Male (49.3–51.5)	2 years: 778 (8.4%)/9279; 3 years: 578 (9.4)/6153; 4 years: 624 (15.1%)/4142; 5 years: 672 (16.2%)/4148; 7 years: 787 (22.0%)/3583	FRI	2–4 years, 5 years, 7 years
Shimada et al. [32]	Japan (NR)	Cross‐sectional	Nursing home	Mean ± SD: 82.6 ± 7.4	Female (70.0), Male (30.0)	188 (3.7%)/5062	Fall‐related fracture	1 year (in the past)
Song et al. [33]	USA (2015–2019)	Case‐control	Hospital	NR	NR	1000 (33.3%)/3000	FRI	6 months, 1 year
Speiser et al. [34]	USA (2010–2013)	Prospective cohort	Community	Mean ± SD: 78.8 ± 5.2 (Training data), 79.0 ± 5.2 (Testing data)	Female (67.2), Male (32.8)	159 (9.7%)/1635	FRI	2 years
Taseh et al. [35]	USA (NR)	Case‐control	Hospital	Median (Q1, Q3): 74 (67, 80) (Fracture group), 72 (62, 83) (Nonfracture group)	Female (64.4), Male (35.6)	261 (50%)/522	Fall‐related hip fracture	NR
Zhao et al. [36]	China (2014–2018)	Case‐control	Hospital	Mean ± SD: 78.2 ± 9.0	Female (45.5), Male (54.5)	115 (33.3%)/345	FRI	During hospitalization

*Note:* USA, United States; Q1, first quartile; Q3, third quartile; IQR, interquartile range.

Abbreviations: FRI, fall‐related injuries; NR, not reported; SD, standard deviation; UK, United Kingdom.

Regarding outcomes and predictors, two studies were specifically concerned with fall‐related fractures [32, 35], and one study focused on fall‐related cervical spine injuries [27]. All outcome measures were binary, with three exclusively involving fallers [24, 27, 35]. Supporting Information Table 3 contains detailed definitions and categories of outcomes in the included studies.

### 3.3. Characteristics of the Models

Table 2 displays the methods of model development or validation and their performance measures in the included studies. Seven studies were classified as TRIPOD type 1a [22, 24, 26, 29, 31, 32, 35], one study as TRIPOD type 1b [33], four as TRIPOD type 2a [23, 25, 34, 36], two as TRIPOD type 2b [27, 30] and one as TRIPOD type 4 [28]. In the final models, the number of predictors ranged from 4 to 70 across studies, with a total of 123 unique predictors being used. Age (*n* = 10) and sex (*n* = 7) were the most common predictors. The predictors included in each study and their frequency of use is shown in Supporting information Table 4 and Supporting Information Table 5, respectively. Without utilizing any screening methods, four studies directly included predictors that were prespecified [29, 31–33]. The predictive factor screening methods involved in the other studies comprised stepwise selection [22, 26, 35], Lasso regression [23, 25], principal component analysis [24], best subsets regression [27] and LightGBM model [30]. Univariate analysis was used to screen predictors in only one study [36]. Logistic regression was the predominant method used in developing models [23, 24, 31–33, 35, 36].

**TABLE 2:** Methods of model development or validation and their performance measures.

Author (publication year)	TRIPOD study type	Number of candidate predictors (predictors in final model)	Method of variable selection	Method of model development	Discrimination performance in development	Calibration method and narrative	Methods of model validation	Discrimination performance in validation	Model presentation
Chan et al. [22]	Type 1a	33 (15)	Direct inclusion (previously established risk factors), backward stepwise selection (digital gait biomarkers)	Cox	NR	NR	NA	NA	Formula of hazard function, table of risk factors with estimated coefficients
Chen et al. [23]	Type 2a	107 (28)	Lasso regression	LR	NR	Brier score: 0.086	IV: The dataset was randomly divided into training set and testing set with a ratio of 7:3	AUC: 0.757 (0.683–0.820)	SHAP summary plot
Davis et al. [24]	Type 1a	27 (8)	Principal component analysis	LR	NR	NR	NA	NA	Forest plot with the likelihood of FRI
Duprey et al. [25]	Type 2a	232 (2 year risk full model/6 month risk full model: 70; 2 year risk short tool: 5)	Lasso regression	Fine‐Gray	AUC: 0.70 (0.70–0.71) (2 year risk full model), 0.71 (0.70–0.71) (6 month risk full model), 0.67 (0.66–0.67) (2 year risk short tool)	Calibration plot: good	IV: The dataset was randomly divided into training set and testing set with a ratio of 2:1	AUC: 0.70 (0.70–0.71) (2 year risk full model), 0.71 (0.70–0.72) (6 month risk full model)	2 year risk full model: Table of risk factors with estimated coefficients; 6 month risk full model: Table of risk factors with estimated coefficients; 2 year risk short tool: Score chart, table of risk factors with estimated coefficients
Ek et al. [26]	Type 1a	26 (4)	Backward stepwise selection	Cox	Harrell’s C statistics: 0.75 (Female), 0.77 (Male)	NR	NA	NA	Score chart
Engelbart et al. [27]	Type 2b	11 (Model including midline tenderness: 3; Model not including midline tenderness: 2)	Best subsets regression	GLM	AUC: 0.822 (Model including midline tenderness), 0.653 (Model not including midline tenderness)	NR	EV: The dataset was divided into training set (data between January 2016 and July 2017) and testing set (data between January 2012 and December 2015)	AUC: 0.832 (Model including midline tenderness), 0.656 (Model not including midline tenderness)	Table of risk factors with odds ratios
Frisendahl et al. [28]	Type 4	The FIF screening tool with 4 predictors^∗^	NA	NA	NA	Hosmer–Lemeshow test *p* value: 0.10 (female), 0.92 (male) (Cohort 1); 0.60 (female), 0.13 (male) (Cohort 2)	EV: Application of two external datasets	Harrell’s C statistics: 0.73 (female), 0.74 (male) (Cohort 1); 0.72 (Female), 0.89 (Male) (Cohort 2)	NA
Frisendahl et al. [29]	Type 1a	4 (4)	Direct inclusion	Cox	Harrell’s C statistics: 0.70 (Female), 0.71 (Male)	NR	NA	NA	Score chart
Heo et al. [30]	Type 2b	187 (26)	LightGBM model	CatBoost	NR	Calibration plot: good	EV: The dataset was divided into training set (patients from the 2018 database) and testing set (patients from the 2019 database)	AUC: 0.700	SHAP summary plot, SHAP waterfall plot
Li et al. [31]	Type 1a	11 (11)	Direct inclusion	LR	AUC: 0.65 (2 years), 0.66 (3 years), 0.64 (4 years), 0.66 (5 years), 0.64 (7 years)	Cox‐Snell *R* ^2^: 0.024 (2 years), 0.031 (3 years), 0.035 (4 years), 0.045 (5 years), 0.052 (7 years); Nagelkerke *R* ^2^: 0.055 (2 years), 0.067 (3 years), 0.061 (4 years), 0.076 (5 years), 0.079 (7 years)	NA	NA	Table of risk factors with incidence rate ratio
Shimada et al. [32]	Type 1a	13 (13)	Direct inclusion	LR	AUC: 0.69 (0.65–0.73)	Hosmer–Lemeshow test *p* value: 0.12	NA	NA	Table of risk factors with odds ratios
Song et al. [33]	Type 1b	31 (31)	Direct inclusion	LR, SVM, RF, NN	NR	NR	IV: Cross‐validation	AUC: 0.75 (LR), 0.76 (SVM), 0.76 (RF), 0.76 (NN) (6 months); 0.74 (LR), 0.74 (SVM), 0.74 (RF), 0.73 (NN) (1 year)	A fall injury‐prevention clinical decision support implementation prototype to link patient‐specific determinants
Speiser et al. [34]	Type 2a	129 (DT: 7; RF: 10)	NR	DT, RF	AUC: 0.75 (0.65–0.85) (DT), 1.00 (0.99–1.00) (RF)	NR	IV: The dataset was randomly divided into training set and testing set with a ratio of 1:1	AUC: 0.54 (0.45–0.62) (DT), 0.66 (0.60–0.71) (RF)	DT model: Figure of eight splitting nodes for seven predictors; RF model: Partial dependence plots based on the Gini criterion for ten predictors
Taseh et al. [35]	Type 1a	12 (4)	Stepwise selection	LR	AUC: 0.67	NR	NA	NA	Online score calculation program
Zhao et al. [36]	Type 2a	18 (5)	Univariate analysis	CLR	AUC: 0.88 (0.85–0.91)	Calibration plot: good	IV: The dataset was randomly divided into training set and testing set with a ratio of 7:3	AUC: 0.847 (0.771–0.924)	Nomogram

*Note:* TRIPOD, Transparent Reporting of a Multivariable Prediction Model for Individual Prognosis or Diagnosis; Cox, Cox proportional hazards model; Fine‐Gray, fine‐gray subdistribution hazards regression; AUC, area under the receiver operating characteristic curve.

Abbreviations: CLR, conditional logistic regression; DT, decision tree; EV, external validation; FRI, fall‐related injuries; GLM, generalized linear model; IV, internal validation; LR, logistic regression; NA, not applicable; NN, neural network; NR, not reported; RF, random forest; SVM, support vector machine.

^∗^The study externally validated the existing tool.

Several studies developed multiple models resulting in a total of 31 multivariable prediction models, with five undergoing external validation. The area under the receiver operating characteristic curve (AUC) was the most frequently reported discrimination statistic, with Harrell’s C statistic following. The discrimination statistic was reported for 19 models in studies that developed one or more models, with values between 0.64 and 1.00, and 12 models did not report confidence intervals for the point estimate. A prospective cohort study from the United States, using a random forest algorithm, emerged as the best‐performing model during the development stage [34]. Discrimination statistics for internal validation were reported in only five studies [23, 25, 33, 34, 36]. The discrimination statistics for 14 models during internal validation ranged from 0.54 to 0.847. The model that excelled in internal validation was a case‐control study from China, which used conditional logistic regression [36]. Five models underwent external validation in three studies [27, 28, 30], with discrimination values ranging from 0.656 to 0.89. The best‐performing model was validated in a group of men for predicting first‐time FRI [28]. Out of all the studies, only seven examined calibration, typically employing the calibration plot and Hosmer–Lemeshow test. A table listing risk factors along with estimated coefficients was the most prevalent model presentation method, employed by five studies [22, 25, 27, 31, 32].

### 3.4. Results of Quality Assessment

**FIGURE 2:**
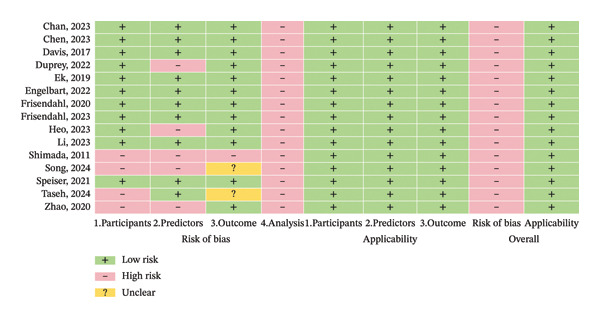
PROBAST results of the included studies.

**FIGURE 3:**
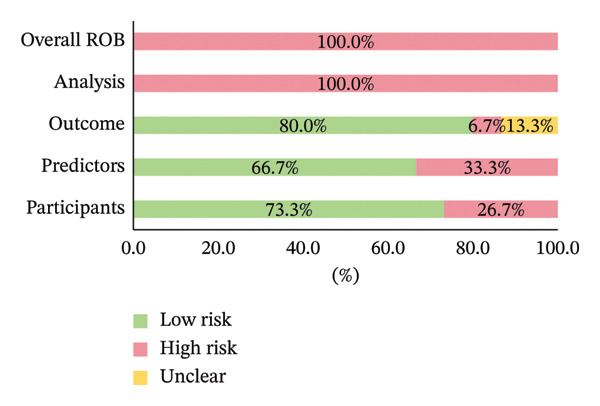
ROB assessment for included studies.

An assessment of bias risk and applicability for the included studies was conducted using the PROBAST tool. All 15 studies were judged to have low concerns regarding applicability. However, all studies (100%) were rated as having a high overall risk of bias (Figure 2). As shown in Figure 3, the proportions of studies with high bias risk in different domains were as follows: participants’ domain (26.7%), predictors’ domain (33.3%), outcome domain (6.7%), and analysis domain (100.0%). In the participants’ domain, four studies were judged to have a high risk of bias chiefly due to the use of improper data sources [32, 33, 35, 36]. In the predictors’ domain, five studies were judged at high risk, often related to possible variations in the definition and measurement of the same predictor [25, 30, 33, 36]. In the outcome domain, two studies had unclear risk due to insufficient reporting of outcome definitions [32, 33]. One study was judged at high risk due to the fact that the time interval between predictor evaluation and outcome relied on retrospective falls [32]. In the analysis domain, the specific methodological issues leading to the high risk‐of‐bias judgments across studies included a lack of optimism correction (86.7% of studies) [22–27, 29–32, 34–36], not including all enrolled participants in the analysis (60.0%) [23–26, 28, 29, 31–33], inappropriate handling of missing data (53.3%) [23–25, 28, 29, 31–33], and having an insufficient number of events per predictor variable (EPV) (40.0%) [23, 24, 26, 27, 34, 36]. Additionally, numerous studies did not provide sufficient details about whether data complexity was addressed [22–25, 27, 29–35] and if the models were properly evaluated [22, 24, 26, 27, 29, 33–35]. The detailed PROBAST domain assessment results for risk of bias are displayed in Supporting Information Figure 1.

The adherence to the TRIPOD + AI checklist for each study is shown in Supporting Information Table 6. The TRIPOD + AI checklist had a median adherence of 56.4% (range: 31.3%–71.4%), whereas for TRIPOD + AI Abstracts, the median was 58.4% (range: 30.8%–92.3%). The items with the lowest adherence rates were related to the lack of details regarding sample size calculation, model heterogeneity across clusters, availability of analytical codes, involvement of patients and the public, and model usability. Supporting Information Figure 2 and Supporting Information Figure 3 provided the assessment of adherence to the TRIPOD + AI guidelines across sections and items.

## 4. Discussion

### 4.1. Summary of Main Findings

This systematic review identified and critically appraised 15 studies reporting 31 multivariable prediction models for FRI in older adults. The discriminative performance of 19 models was evaluated in eight studies, revealing poor to good outcomes in internal or external validation, with statistical values ranging from 0.54 to 0.89. The findings indicated significant variations among the different prediction models for this purpose, suggesting that healthcare workers should proceed with caution in their application.

Based on the PROBAST checklist, most studies had sufficiently clear descriptions of participants, predictors, and outcomes to avoid bias. However, all studies were regarded as having a high risk of bias, largely due to inadequate analysis. Four key methodological flaws in the analysis domain were identified. First, six studies had fewer than 10 EPV during model development, resulting in a high risk of bias via potential overfitting and skewed performance estimates [16]. Second, except for three studies that employed sensitivity analysis [26, 37] or the standard random forest imputation method [34] to address missing data and assess its impact, most studies excluded participants with missing data, introducing potential selection bias and less accurate predictions [38]. Third, five studies conducted internal validation, mostly via random data splitting (*n* = 4), which was demonstrated to underutilize data and yield overly negative performance assessments [39, 40]. While Song et al. [33] provided a reassuring example by fine‐tuning models through cross‐validation to pick the best parameters and testing their performance on an independent set to tackle overfitting. Fourth, most studies on model development lacked proper validation and detailed performance reporting. This increases the likelihood of bias in the study and also weakens the trustworthiness of the model’s performance, affecting how healthcare workers judge and choose the model, as an excess of models without proper validation is not helpful in clinical practice [41, 42]. Additionally, all studies failed to adhere to TRIPOD + AI guidelines, leading to transparency gaps and potential bias risks.

### 4.2. Comparison With Existing Reviews

To our knowledge, this study represents the first systematic review of multivariable prediction models for FRI in older adults. Previous systematic reviews in the related field have primarily concentrated on models for predicting falls. Existing reviews for predicting falls across different settings, including hospitalized patients [43] and community‐dwelling older adults [6, 7], uniformly reported an abundance of developed models that were severely limited by high risk of bias and inadequate validation. Our review also identified this central methodological critique, identifying similar prevalent flaws in the analysis domain. Furthermore, our review extends the literature by shifting the focus of the predictions from the event of a fall to its clinically consequential outcome—injury, which helps to provide guidance for healthcare providers in implementing more targeted intervention strategies, thereby optimizing resource utilization and reducing the adverse consequences associated with falls.

### 4.3. Implications for Clinical Practice and Technology Development

For clinical practice, most included studies employed inconsistent definitions for FRI. For instance, some studies have defined FRI as those necessitating medical treatment [23, 31], whereas others have characterized them as falls resulting in fractures or hospital admissions [22, 28, 34]. Future clinical applications should prioritize models with standardized FRI definitions to ensure consistent risk assessment. Furthermore, while age and sex are consistently identified as key predictors, the clinical actionability of many other variables remains unclear due to high variability across models. This underscores the need for clinical judgment to contextualize risk assessment within individual patient profiles, including factors like functional status.

From a technology development perspective, this review observed a predominant use of traditional logistic regression among the included studies. However, one study showed that a CatBoost algorithm, a machine learning technology, outperformed a logistic regression model in predicting serious FRI [30], suggesting a potential path to higher accuracy. A critical constraint identified for such advanced methods, consistent with broader literature, is their frequent lack of model transparency and interpretability [44], which hinders clinical adoption. In the future, efforts should be made to develop and promote the adoption of explainable machine learning technologies in predictive model construction.

### 4.4. Methodological Limitations

This study has several limitations as well. First, regarding search strategy and study screening, our inclusion was restricted to English‐language publications, and gray literature was not consulted. This may have introduced language and publication bias, potentially omitting relevant studies. Second, the studies included lacked sufficient numbers of external validation models (none of the models were validated in more than five independent cohorts), which prevented meaningful quantitative synthesis or meta‐analysis. Our findings were therefore limited to a qualitative summary.

### 4.5. Future Research Directions

Future research should prioritize the following directions to advance the FRI prediction in older adults. First, most prior studies have categorized FRI as a binary variable, failing to differentiate between varying degrees of severity. Future efforts should focus on developing risk stratification prediction models capable of differentiating FRI severity. This process should incorporate interpretable artificial intelligence techniques to ensure these complex models are clinically acceptable and actionable. Second, future prospective model development studies must ensure a steadfast commitment to methodological rigor, such as adequate sample size, appropriate strategies for handling missing data, and robust internal validation techniques like bootstrapping or cross‐validation rather than simple data splitting. Third, it is recommended that newly developed models undergo rigorous internal and external validation in diverse, representative populations. Researchers should adhere to reporting guidelines such as TRIPOD + AI, providing complete details to enable independent validation, replication, and support eventual clinical implementation.

## 5. Conclusions

The systematic review comprising 31 models from 15 studies revealed substantial discrimination variability among the prediction models for FRI in older adults. Crucially, all models had a high risk of bias, primarily in the analysis domain. Therefore, no existing model is currently suitable for clinical implementation. Future research must shift focus from development alone to methodological rigor. Priorities include using adequate samples, robust internal validation techniques, and external validation in diverse populations. Adherence to TRIPOD + AI and PROBAST guidelines is essential for critical appraisal and replication. Once an evidence‐based model is available, efforts to disseminate it and implement it into clinical practice are recommended.

## Funding

This study was supported by National Key Research and Development Program of China (2023YFC3605900).

## Disclosure

The funding agency did not involve in the study design, data collection, data analysis and interpretation, the writing of this manuscript, or the decision to submit the article for publication.

## Conflicts of Interest

The authors declare no conflicts of interest.

## Supporting Information

Supporting Information Table 1. Search strategy in different databases.

Supporting Information Table 2. Studies excluded during full‐text screening (*n* = 162).

Supporting Information Table 3. Definition and categories of outcome in the included studies.

Supporting Information Table 4. Predictors in the final prediction model of the included studies.

Supporting Information Table 5. Frequency of predictors in the included studies and their respective proportions.

Supporting Information Table 6. Overall adherence to the TRIPOD + AI of the included studies.

Supporting Information Figure 1. Detailed PROBAST domain assessment results for risk of bias.

Supporting Information Figure 2. Adherence to the TRIPOD + AI for Abstracts checklist across the items.

Supporting Information Figure 3. Adherence to the TRIPOD + AI checklist across the items.

## Supporting information


**Supporting Information** Additional supporting information can be found online in the Supporting Information section.

## Data Availability

Data sharing is not applicable to this study as no datasets were generated during the current study.
