# Supplementary material for: A Systematic Review of Multivariate Models for Predicting Fall‐Related Injuries in Older Adults
Source: J Nurs Manag. 2026 Mar 24;2026:1740588. doi: 10.1155/jonm/1740588 (PMC13140816; doi:10.1155/jonm/1740588)
Supplement: Supplementary file 1 — Supporting Information Additional supporting information can be found online in the Supporting Information section. [file JONM-2026-1740588-s001.docx]

**Supplementary Materials**

# Supplementary material table 1. Search strategy in different databases

| **Database** | **Search strategy** | **Search date** |
| --- | --- | --- |
| Medline (via OVID) | 1 exp Accidental Falls/ OR fall*.ti,ab.  2 exp "Wounds and Injuries"/ OR exp Fractures, Bone/ OR (injur* OR fracture*).ti,ab.  3 1 AND 2  4 predict*.ti.  5 (predict* AND (outcome* OR risk* OR model*)).mp.  6 ((variable* OR criteria OR scor* OR factor*) AND (predict* OR decision* OR prognos*)).mp.  7 ((decision* OR prognostic) AND model*).mp.  8 decision*.mp. AND Logistic Models/  9 exp ROC Curve/ OR exp Algorithms/  10 ("ROC curve" or "c-statistic" or "c statistic" or "area under the curve" or AUC or algorithm*).mp.  11 4 OR 5 OR 6 OR 7 OR 8 OR 9 OR 10  12 exp Aged/ OR exp Aging/  13 (aged OR aging OR ageing OR elder* OR geriatric* OR gerontolog* OR senile OR senior* OR pensioner* OR "age group*" OR "older adult*" OR "older population*" OR "older people" OR "older patient*" OR "older person*").ti,ab.  14 12 OR 13  15 english.lg.  16 3 AND 11 AND 14 AND 15 | 23 May 2024 |
| Embase (via OVID) | 1 exp falling/ OR fall*.ti,ab.  2 exp injury/ OR exp fracture/ OR (injur* OR fracture*).ti,ab.  3 1 AND 2  4 predict*.ti.  5 (predict* AND (outcome* OR risk* OR model*)).mp.  6 ((variable* OR criteria OR scor* OR factor*) AND (predict* OR decision* OR prognos*)).mp.  7 ((decision* OR prognostic) AND model*).mp.  8 decision*.mp. AND statistical model/  9 exp receiver operating characteristic/ OR exp algorithm/  10 ("ROC curve" OR "c-statistic" OR "c statistic" OR "area under the curve" OR "AUC" OR algorithm*).mp.  11 4 OR 5 OR 6 OR 7 OR 8 OR 9 OR 10  12 exp aged/ OR exp aging/  13 (aged OR aging OR ageing OR elder* OR geriatric* OR gerontolog* OR senile OR senior* OR pensioner* OR "age group*" OR "older adult*" OR "older population*" OR "older people" OR "older patient*" OR "older person*").ti,ab.  14 12 OR 13  15 english.lg.  16 3 AND 11 AND 14 AND 15 | 23 May 2024 |
| Cochrane Library | #1 MeSH descriptor: [Accidental Falls] explode all trees  #2 (fall*):ti,ab,kw  #3 #1 OR #2  #4 MeSH descriptor: [Wounds and Injuries] explode all trees  #5 MeSH descriptor: [Fractures, Bone] explode all trees  #6 (injur* OR fracture*):ti,ab,kw  #7 #4 OR #5 OR #6  #8 #3 AND #7  #9 (predict*):ti  #10 (predict* AND (outcome* OR risk* OR model*)):ti,ab,kw  #11 ((variable* OR criteria OR scor* OR factor*) AND (predict* OR decision* OR prognos*)):ti,ab,kw  #12 ((decision* OR prognostic) AND model*):ti,ab,kw  #13 (decision*):ti,ab,kw  #14 MeSH descriptor: [Logistic Models] explode all trees  #15 #13 AND #14  #16 #9 OR #10 OR #11 OR #12 OR #15  #17 MeSH descriptor: [Aged] explode all trees  #18 (aged OR aging OR ageing OR elder* OR geriatric* OR gerontolog* OR senile OR senior* OR pensioner* OR age NEXT group* OR older NEXT adult* OR older NEXT population* OR older NEXT people OR older NEXT patient* OR older NEXT person*):ti,ab,kw  #19 #17 OR #18  #20 English:la  #21 #8 AND #16 AND #19 AND #20 | 23 May 2024 |
| CINAHL (via EBSCO) | S1 (MH "Accidental Falls") OR TI fall* OR AB fall*  S2 (MH "Wounds and Injuries+") OR (MH "Fractures+") OR TI ( injur* OR fracture* ) OR AB ( injur* OR fracture*)  S3 S1 AND S2  S4 TI predict*  S5 TI ( predict* AND (outcome* OR risk* OR model*) ) OR AB ( predict* AND (outcome* OR risk* OR model*) )  S6 TI ( (variable* OR criteria OR scor* OR factor*) AND (predict* OR decision* OR prognos*) ) OR AB ( (variable* OR criteria OR scor* OR factor*) AND (predict* OR decision* OR prognos*) )  S7 TI ( (decision* OR prognostic) AND model*) OR AB ( (decision* OR prognostic) AND model*)  S8 TI decision* OR AB decision*  S9 (MH "Logistic Regression+")  S10 S8 AND S9  S11 (MH "ROC Curve") OR (MH "Algorithms")  S12 TI ( "ROC curve" OR "c-statistic" OR "c statistic" OR "area under the curve" OR AUC OR algorithm* ) OR AB ( "ROC curve" OR "c-statistic" OR "c statistic" OR "area under the curve" OR AUC OR algorithm* )  S13 S4 OR S5 OR S6 OR S7 OR S10 OR S11 OR S12  S14 (MH "Aged+") OR (MH "Aging")  S15 TI ( aged OR aging OR ageing OR elder* OR geriatric* OR gerontolog* OR senile OR senior* OR pensioner* OR "age group*" OR "older adult*" OR "older population*" OR "older people" OR "older patient*" OR "older person*" ) OR AB ( aged OR aging OR ageing OR elder* OR geriatric* OR gerontolog* OR senile OR senior* OR pensioner* OR "age group*" OR "older adult*" OR "older population*" OR "older people" OR "older patient*" OR "older person*")  S16 S14 OR S15  S17 LA english  S18 S3 AND S13 AND S16 AND S17 | 23 May 2024 |
| Web of Science | #1 TS=(fall* AND (injur* OR fracture*))  #2 TI=(predict*)  #3 TS=(predict* AND (outcome* OR risk* OR model*))  #4 TS=((variable* OR criteria OR scor* OR factor*) AND (predict* OR decision* OR prognos*))  #5 TS=((decision* OR prognostic) AND model*)  #6 TS=("c-statistic" OR "c statistic" OR "area under the curve" OR AUC OR algorithm*)  #7#2 OR #3 OR #4 OR #5 OR #6  #8 TS=(aged OR aging OR ageing OR elder* OR geriatric* OR gerontolog* OR senile OR senior* OR pensioner* OR "age group*" OR "older adult*" OR "older population*" OR "older people" OR "older patient*" OR "older person*")  #9 LA=(English)  #10 #1 AND #7 AND #8 AND #9 | 23 May 2024 |
| Scopus | ( TITLE-ABS-KEY ( fall* AND ( injur* OR fracture* ) ) ) AND ( ( TITLE ( predict* ) OR TITLE-ABS-KEY ( predict* AND ( outcome* OR risk* OR model* ) ) OR TITLE-ABS-KEY ( ( variable* OR criteria OR scor* OR factor* ) AND ( predict* OR decision* OR prognos* ) ) OR TITLE-ABS-KEY ( ( decision* OR prognostic ) AND model* ) OR TITLE-ABS-KEY ( "roc curve" OR "c-statistic" OR "c statistic" OR "area under the curve" OR auc OR algorithm* ) ) ) AND ( TITLE-ABS-KEY ( aged OR aging OR ageing OR elder* OR geriatric* OR gerontolog* OR senile OR senior* OR pensioner* OR "age group*" OR "older adult*" OR "older population*" OR "older people" OR "older patient*" OR "older person*" ) ) AND ( LANGUAGE ( english ) ) | 23 May 2024 |

# Supplementary material table 2. Studies excluded during full-text screening (*n* = 162)

| Study information | Reason of exclusion |
| --- | --- |
| Lin CC, et al. The Predictive Validity and Clinical Application of Stopping Elderly Accidents, Deaths & Injuries (STEADI) for Fall Risk Screening. Advances in Geriatric Medicine and Research 2022;4(3):e220008. | Review papers |
| Masud T, et al. Official Positions for FRAX R clinical regarding falls and frailty: can falls and frailty be used in FRAX R? . Journal of Clinical Densitometry 2011;14(3):194-204. | Review papers |
| Milic J, et al. Moving from the prediction of fractures to the prediction of falls in an aging HIV scenario. Aids 2023;37(9):1467-1469. | Review papers |
| Tinetti ME. Prevention of Falls and Fall Injuries in Elderly Persons: A Research Agenda. Preventive Medicine 1994;23(5):756-762. | Review papers |
| Young D, et al. Falls risk assessment and management system (FRAMS) - A decision support tool developed for general practitioners and their primary care team. Hong Kong Practitioner 2005;27:306-310. | Review papers |
| Martinez MC, et al. Validity and reliability of the Brazilian version of the Johns Hopkins Fall Risk Assessment Tool to assess the risk of falls. Rev Bras Epidemiol 2019;22:e190037. | Non-English papers |
| Adams JM, et al. Does patient age and height of fall alone require trauma team activation? Am Surg 2011;77(9):1201-1205. | Didn’t include fall-related injury as an outcome |
| Adeyemi O, et al. Diagnostic Accuracy and Risk Stratification of a Trauma Risk Assessment Tool Among those with Fall Injuries. medRxiv 2023;45(6):340-351. | Didn’t include fall-related injury as an outcome |
| Ahmed L, et al. External validation of the garvan nomograms for predicting absolute fracture risk: The tromso study. J Bone Miner Res 2014;9(9):e107695. | Didn’t include fall-related injury as an outcome |
| Altai Z, et al. The effect of boundary and loading conditions on patient classification using finite element predicted risk of fracture. Clinical Biomechanics 2019;68:137-143. | Didn’t include fall-related injury as an outcome |
| Archer L, et al. Development and external validation of the eFalls tool: a multivariable prediction model for the risk of ED attendance or hospitalisation with a fall or fracture in older adults. Age & Ageing 2024;53:afae057. | Didn’t include fall-related injury as an outcome |
| Baus A, et al. Developing Methods of Repurposing Electronic Health Record Data for Identification of Older Adults at Risk of Unintentional Falls. Perspectives in Health Information Management 2016;13(Spring):1b. | Didn’t include fall-related injury as an outcome |
| Bruce J, et al. Falls and fracture risk screening in primary care: update and validation of a postal screening tool for community dwelling older adults recruited to UK Prevention of Falls Injury Trial (PreFIT). BMC Geriatrics 2023;23(1):42. | Didn’t include fall-related injury as an outcome |
| Chen XD, et al. Long-term trajectories of depressive symptoms and machine learning techniques for fall prediction in older adults: Evidence from the China Health and Retirement Longitudinal Study (CHARLS). Arch Gerontol Geriatr 2023;111:105012. | Didn’t include fall-related injury as an outcome |
| Chen YH, et al. Applying artificial intelligence to predict falls for inpatient. Frontiers in Medicine 2023;10:1285192. | Didn’t include fall-related injury as an outcome |
| Cheng ZN, et al. Incidence of accidental falls and development of a fall risk prediction model among elderly patients with diabetes mellitus: A prospective cohort study. Journal of Clinical Nursing 2023;32(7-8):1398-1409. | Didn’t include fall-related injury as an outcome |
| Choi Y, et al. A dynamic risk model for inpatient falls. American Journal of Health-System Pharmacy 2018;75(17):1293-1303. | Didn’t include fall-related injury as an outcome |
| Cleary K, et al. Predicting falls in community dwelling older adults using the Activities-specific Balance Confidence Scale. Archives of Gerontology & Geriatrics 2017;72:142-145. | Didn’t include fall-related injury as an outcome |
| Coster ME, et al. Physical function tests predict incident falls: A prospective study of 2969 men in the Swedish Osteoporotic Fractures in Men study. Scandinavian Journal of Public Health 2020;48(4):436-441. | Didn’t include fall-related injury as an outcome |
| Crow RS, et al. Frailty Versus Stopping Elderly Accidents, Deaths and Injuries Initiative Fall Risk Score: Ability to Predict Future Falls. Journal of the American Geriatrics Society 2018;66(3):577-583. | Didn’t include fall-related injury as an outcome |
| Cuaya G, et al. A dynamic Bayesian network for estimating the risk of falls from real gait data. Medical & Biological Engineering & Computing 2013;51(1-2):29-37. | Didn’t include fall-related injury as an outcome |
| Cuaya-Simbro G, et al. Comparison of Machine Learning Models to Predict Risk of Falling in Osteoporosis Elderly. Foundations of Computing and Decision Sciences 2020;45(2):65-77. | Didn’t include fall-related injury as an outcome |
| Delbari A, et al. Association of Home Falls and Accidents Screening Tool (HOME FAST) with risk of fall in older adults: Ardakan Cohort Study on Ageing (ACSA). International Journal of Healthcare Management 2023:1-9. | Didn’t include fall-related injury as an outcome |
| Dormosh N, et al. Development and Internal Validation of a Risk Prediction Model for Falls Among Older People Using Primary Care Electronic Health Records. Journals of Gerontology Series a-Biological Sciences and Medical Sciences 2022;77(7):1438-1445. | Didn’t include fall-related injury as an outcome |
| Fiedorová I, et al. Receiver Operating Characteristic Curve Analysis of the Somatosensory Organization Test, Berg Balance Scale, and Fall Efficacy Scale–International for Predicting Falls in Discharged Stroke Patients. Int J Environ Res Public Health 2022;19(15):9181. | Didn’t include fall-related injury as an outcome |
| Fischer BL, et al. Performance-based assessment of falls risk in older veterans with executive dysfunction. Journal of Rehabilitation Research & Development 2014;5(2):263-274. | Didn’t include fall-related injury as an outcome |
| Forth KE, et al. A Postural Assessment Utilizing Machine Learning Prospectively Identifies Older Adults at a High Risk of Falling. Frontiers in Medicine 2020;7:591517. | Didn’t include fall-related injury as an outcome |
| Fusco-Gessick B, et al. Using Functional Independence Measure Subscales to Predict Falls - Rapid Assessment. Rehabilitation Nursing 2019;44(4):236-244. | Didn’t include fall-related injury as an outcome |
| Gafner SC, et al. The Role of Hip Abductor Strength in Identifying Older Persons at Risk of Falls: A Diagnostic Accuracy Study. Clinical Interventions in Aging 2020;15:645-654. | Didn’t include fall-related injury as an outcome |
| Ge J, et al. A New Index Based on Serum Creatinine and Cystatin C Can Predict the Risks of Sarcopenia, Falls and Fractures in Old Patients with Low Bone Mineral Density. Nutrients 2022;14(23):5020. | Didn’t include fall-related injury as an outcome |
| Gietzelt M, et al. Predicting falls in people with dementia using accelerometry - A one-year prospective multi-center field study. Biomedical Engineering-Biomedizinische Technik 2014;59:S683-+. | Didn’t include fall-related injury as an outcome |
| Girardi M, et al. Predicting fall risks in an elderly population: Computer dynamic posturography versus electronystagmography test results. Laryngoscope 2001;111(9):1528-1532. | Didn’t include fall-related injury as an outcome |
| Greene BR, et al. Evaluation of falls risk in community-dwelling older adults using body-worn sensors. Gerontology 2012;58(5):472-480. | Didn’t include fall-related injury as an outcome |
| Greene BR, et al. Fall Risk Assessment Through Automatic Combination of Clinical Fall Risk Factors and Body-Worn Sensor Data. IEEE Journal of Biomedical & Health Informatics 2017;21(3):725-731. | Didn’t include fall-related injury as an outcome |
| Hartley P, et al. The use of the World Guidelines for Falls Prevention and Management's risk stratification algorithm in predicting falls in The Irish Longitudinal Study on Ageing (TILDA). Age & Ageing 2023;52(7):afad129. | Didn’t include fall-related injury as an outcome |
| Harvey NC, et al. Predictive Value of DXA Appendicular Lean Mass for Incident Fractures, Falls, and Mortality, Independent of Prior Falls, FRAX, and BMD: findings from the Women's Health Initiative (WHI). J Bone Miner Res 2021;36(4):654-661. | Didn’t include fall-related injury as an outcome |
| Hirata R, et al. History of Falls and Bedriddenness Ranks are Useful Predictive Factors for in-Hospital Falls: A Single-Center Retrospective Observational Study the Fall Risk Model. International Journal of General Medicine 2022;15:8121-8131. | Didn’t include fall-related injury as an outcome |
| Hsieh KL, et al. Smartphone technology can measure postural stability and discriminate fall risk in older adults. Gait & Posture 2019;67:160-165. | Didn’t include fall-related injury as an outcome |
| Hsu YC, et al. A Novel Approach for Fall Risk Prediction Using the Inertial Sensor Data From the Timed-Up-and-Go Test in a Community Setting. Ieee Sensors Journal 2020;20(16):9339-9350. | Didn’t include fall-related injury as an outcome |
| Ickert EC, et al. Overestimation of Balance Ability Among Older Adults at Risk for Falls. J Aging Health 2024;36(5-6):286-298. | Didn’t include fall-related injury as an outcome |
| Kannoth S, et al. Adapted Stopping Elderly Accidents, Deaths, and Injuries Questions for Falls Risk Screening: Predictive Ability in Older Drivers. American Journal of Preventive Medicine 2021;61(1):105-114. | Didn’t include fall-related injury as an outcome |
| Kitcharanant N, et al. Validity and reliability of the self-rated fall risk questionnaire in older adults with osteoporosis. BMC Musculoskeletal Disorders 2020;21(1):757. | Didn’t include fall-related injury as an outcome |
| Kiyoshige Y, et al. Fatty degeneration of gluteus minimus muscle as a predictor of falls. Archives of Gerontology & Geriatrics 2015;60(1):59-61. | Didn’t include fall-related injury as an outcome |
| Koshmak G, et al. Dynamic Bayesian networks for context-aware fall risk assessment. Sensors 9330;14(5):9330-9348. | Didn’t include fall-related injury as an outcome |
| Kus B, et al. Comparison of three fall risk assessment tools in older hospitalized patients in Turkey: analysis of sensitivity and specificity. Aging Clin Exp Res 2023;35(5):1033-1041. | Didn’t include fall-related injury as an outcome |
| Lafontant K, et al. Comparing Sensitivity, Specificity, and Accuracy of Fall Risk Assessments in Community-Dwelling Older Adults. Clinical Interventions In Aging 2024;19:581-588. | Didn’t include fall-related injury as an outcome |
| Lage I, et al. Older People Living Alone: A Predictive Model of Fall Risk. Int J Environ Res Public Health 2023;20(13). | Didn’t include fall-related injury as an outcome |
| Lamb SE, et al. The Optimal Sequence and Selection of Screening Test Items to Predict Fall Risk in Older Disabled Women: The Women's Health and Aging Study. Journals of Gerontology Series a-Biological Sciences and Medical Sciences 2008;63(10):1082-1088. | Didn’t include fall-related injury as an outcome |
| Lavanya S, et al. A study on association between fall risk in elderly and frax score. Int J Acad Med Pharm 2023;5(6):27-32. | Didn’t include fall-related injury as an outcome |
| Leclerc BS, et al. Risk factors for falling among community-dwelling seniors using home-care services: An extended hazards model with time-dependent covariates and multiple events. Chronic Diseases in Canada 2008;28(4):111-120. | Didn’t include fall-related injury as an outcome |
| Leslie WD, et al. Fracture prediction from self-reported falls in routine clinical practice: a registry-based cohort study. Osteoporosis International 2019;30(11):2195-2203. | Didn’t include fall-related injury as an outcome |
| Li G, et al. Frailty index of deficit accumulation and falls: data from the Global Longitudinal Study of Osteoporosis in Women (GLOW) Hamilton cohort. BMC Musculoskeletal Disorders 2014;15:185. | Didn’t include fall-related injury as an outcome |
| Li G, et al. Comparison between frailty index of deficit accumulation and phenotypic model to predict risk of falls: data from the global longitudinal study of osteoporosis in women (GLOW) Hamilton cohort. PLoS ONE 2015;10(3):e0120144. | Didn’t include fall-related injury as an outcome |
| Liang CK, et al. Gait speed and risk assessment for falls among men aged 80 years and older: A prospective cohort study in Taiwan. Eur Geriatr Med 2014;5(5):298-302. | Didn’t include fall-related injury as an outcome |
| Lindberg DS, et al. Identification of important factors in an inpatient fall risk prediction model to improve the quality of care using EHR and electronic administrative data: A machine-learning approach. International Journal of Medical Informatics 2020;143:104272. | Didn’t include fall-related injury as an outcome |
| Lindemann U, et al. Maximum step length as a potential screening tool for falls in non-disabled older adults living in the community. Aging Clin Exp Res 2008;20(5):394-399. | Didn’t include fall-related injury as an outcome |
| Lo Y, et al. Using Machine Learning on Home Health Care Assessments to Predict Fall Risk. Studies in Health Technology & Informatics 2019;264:684-688. | Didn’t include fall-related injury as an outcome |
| Lohman MC, et al. Operationalisation and validation of the Stopping Elderly Accidents, Deaths, and Injuries (STEADI) fall risk algorithm in a nationally representative sample. J Epidemiol Community Health 2017;71(12):1191-1197. | Didn’t include fall-related injury as an outcome |
| Lyu ZY, et al. The Identification of Elderly People with High Fall Risk Using Machine Learning Algorithms. Healthcare 2023;11(1):47. | Didn’t include fall-related injury as an outcome |
| Marschollek M, et al. Sensor-based Fall Risk Assessment - an Expert 'to go'. Methods of Information in Medicine 2011;50(5):420-426. | Didn’t include fall-related injury as an outcome |
| Martincová A, et al. Predictive Model of the Risk of Fall Based on Physical Fitness Assessment in Older Adults. Studia Sportiva 2023;17(2):141-150. | Didn’t include fall-related injury as an outcome |
| Mielenz TJ, et al. Evaluating a Two-Level vs. Three-Level Fall Risk Screening Algorithm for Predicting Falls Among Older Adults. Frontiers in Public Health 2020;8:373. | Didn’t include fall-related injury as an outcome |
| Milisen K, et al. Fall prediction in inpatients by bedside nurses using the St. Thomas's risk assessment tool in falling elderly inpatients (STRATIFY) instrument: A multicenter study. Journal of the American Geriatrics Society 2007;55(5):725-733. | Didn’t include fall-related injury as an outcome |
| Mishra AK, et al. Explainable Fall Risk Prediction in Older Adults Using Gait and Geriatric Assessments. Frontiers in Digital Health 2022;4:869812. | Didn’t include fall-related injury as an outcome |
| Muhaidat J, et al. Validity of Simple Gait-Related Dual-Task Tests in Predicting Falls in Community-Dwelling Older Adults. Arch Phys Med Rehabil 2014;95(1):58-64. | Didn’t include fall-related injury as an outcome |
| Nanda S, et al. Fall Risk Assessment in Geriatric-Psychiatric Inpatients to Lower Events (FRAGILE). Journal of Gerontological Nursing 2011;37(2):22-30. | Didn’t include fall-related injury as an outcome |
| Nandy S, et al. Development and preliminary examination of the predictive validity of the Falls Risk Assessment Tool (FRAT) for use in primary care. Journal of Public Health 2004;26(2):138-143. | Didn’t include fall-related injury as an outcome |
| Neyens J, et al. The development of a multidisciplinary fall risk evaluation tool for demented nursing home patients in the Netherlands. BMC Public Health 2006;6:74. | Didn’t include fall-related injury as an outcome |
| Nishiyama D, et al. Accurate fall risk classification in elderly using one gait cycle data and machine learning. Clinical Biomechanics 2024;115:106262. | Didn’t include fall-related injury as an outcome |
| Pereira A, et al. The Mini-Balance Evaluation System Test Can Predict Falls in Clinically Stable Outpatients With COPD: A 12-MO PROSPECTIVE COHORT STUDY. Journal of Cardiopulmonary Rehabilitation & Prevention 2019;39(6):391-396. | Didn’t include fall-related injury as an outcome |
| Poe SS, et al. The Johns Hopkins Fall Risk Assessment Tool: A Study of Reliability and Validity. Journal of Nursing Care Quality 2018;33(1):10-19. | Didn’t include fall-related injury as an outcome |
| Roe L, et al. Breaking Up Sedentary Time Reduces Recurrent Fall Risk, but Not Incident Fracture Risk in Older Men. JBMR Plus 2023;7(12):e10803. | Didn’t include fall-related injury as an outcome |
| Rosario E, et al. Casa Colina Fall Risk Assessment Scale-Revised: Predicting Falls in Inpatient Rehabilitation Facilities. Archives of Rehabilitation Research and Clinical Translation 2022;4(4):100233. | Didn’t include fall-related injury as an outcome |
| Roshdibenam V, et al. Machine Learning Prediction of Fall Risk in Older Adults Using Timed Up and Go Test Kinematics. Sensors 2021;21(10):3481. | Didn’t include fall-related injury as an outcome |
| Samah ZA, et al. Discriminative and predictive ability of physical performance measures in identifying fall risk among older adults. Sains Malaysiana 2018;47(11):2769-2776. | Didn’t include fall-related injury as an outcome |
| Satoh M, et al. Development and evaluation of a simple predictive model for falls in acute care setting. Journal of Clinical Nursing 2023;32(17-18):6474-6484. | Didn’t include fall-related injury as an outcome |
| Schoufour J, et al. The use of a frailty index to predict adverse health outcomes (falls, fractures, hospitalization, medication use, comorbid conditions) in people with intellectual disabilities. Research in Developmental Disabilities 2015;38:39-47. | Didn’t include fall-related injury as an outcome |
| Shee AW, et al. Comparison of two fall risk assessment tools (FRATs) targeting falls prevention in sub-acute care. Arch Gerontol Geriatr 2012;55(3):653-659. | Didn’t include fall-related injury as an outcome |
| Shinoda Y, et al. Prediction of the pathological fracture risk during stance and fall-loading configurations for metastases in the proximal femur, using a computed tomography-based finite element method. Journal of Orthopaedic Science 2019;24(6):1074-1080. | Didn’t include fall-related injury as an outcome |
| Srisim K, et al. Functional assessments for predicting a risk of multiple falls in independent ambulatory patients with spinal cord injury. Journal of Spinal Cord Medicine 2015;38(4):439-445. | Didn’t include fall-related injury as an outcome |
| Tada M, et al. Relationships of the stand-up time to falls and fractures in patients with rheumatoid arthritis: Results from the CHIKARA study. International Journal of Rheumatic Diseases 2021;24(2):246-253. | Didn’t include fall-related injury as an outcome |
| Tago M, et al. Validation and Improvement of the Saga Fall Risk Model: A Multicenter Retrospective Observational Study. Clinical Interventions in Aging 2024;19:175-188. | Didn’t include fall-related injury as an outcome |
| Tago M, et al. New predictive models for falls among inpatients using public ADL scale in Japan: A retrospective observational study of 7,858 patients in acute care setting. PLoS ONE 2020;15(7):e0236130. | Didn’t include fall-related injury as an outcome |
| Tang YT, et al. Using Explainable AI (XAI) for the Prediction of Falls in the Older Population. Algorithms 2022;15(10):353. | Didn’t include fall-related injury as an outcome |
| Thapa R, et al. Predicting Falls in Long-term Care Facilities: Machine Learning Study. Jmir Aging 2022;5(2):e35373 | Didn’t include fall-related injury as an outcome |
| Thorbahn LDB, et al. Use of the Berg balance test to predict falls in elderly persons. Physical Therapy 1996;76(6):576-583. | Didn’t include fall-related injury as an outcome |
| Todorov G, et al. Comparison of fracture risk calculators in elderly fallers: a hospital-based cross-sectional study. BMJ Open 2022;12(7):e060282. | Didn’t include fall-related injury as an outcome |
| Wihlborg A, et al. Fracture predictive ability of physical performance tests and history of falls in elderly women: a 10-year prospective study. Osteoporosis International 2015;26(8):2101-2109. | Didn’t include fall-related injury as an outcome |
| Williams DR, et al. Predictors of falls and fractures in bradykinetic rigid syndromes: a retrospective study. Journal of Neurology, Neurosurgery & Psychiatry 2006;77(4):468-473. | Didn’t include fall-related injury as an outcome |
| Wright J, et al. Designing a Fall Prediction Model for Inpatient Rehabilitation Facilities Using Readily Available Data. Arch Phys Med Rehabil 2024;105(4):704-709. | Didn’t include fall-related injury as an outcome |
| Yamada M, et al. Development of a New Fall Risk Assessment Index for Older Adults. Int J Gerontol 2012;6(3):160-162. | Didn’t include fall-related injury as an outcome |
| Yauk S, et al. Predicting in-hospital falls: development of the Scott and White Falls Risk Screener. Journal of Nursing Care Quality 2005;20(2):128-133. | Didn’t include fall-related injury as an outcome |
| Zaslavsky O, et al. Comparison of Frailty Phenotypes for Prediction of Mortality, Incident Falls, and Hip Fracture in Older Women. Journal of the American Geriatrics Society 2016;64(9):1858-1862. | Didn’t include fall-related injury as an outcome |
| Zaslavsky O, et al. Comparison of the Simplified sWHI and the Standard CHS Frailty Phenotypes for Prediction of Mortality, Incident Falls, and Hip Fractures in Older Women. Journals of Gerontology Series a-Biological Sciences and Medical Sciences 2017;72(10):1394-1400. | Didn’t include fall-related injury as an outcome |
| Beghi E, et al. Prediction of Falls in Subjects Suffering From Parkinson Disease, Multiple Sclerosis, and Stroke. Archives of Physical Medicine & Rehabilitation 2018;99(4):641-651. | Didn’t develop or validate a prediction model |
| Bergland A, et al. Risk factors for serious fall related injury in elderly women living at home. Injury Prevention 2004;10(5):308-313. | Didn’t develop or validate a prediction model |
| Bradley SM, et al. Predictors of serious injury among hospitalized patients evaluated for falls. Journal of Hospital Medicine (Online) 2010;5(2):63-68. | Didn’t develop or validate a prediction model |
| Cai Y, et al. Chronic Pain and Risk of Injurious Falls in Community-Dwelling Older Adults. Journals of Gerontology Series A Biological Sciences & Medical Sciences 2021;76(9):e179-e186. | Didn’t develop or validate a prediction model |
| Chari S, et al. Predictors of fracture from falls reported in hospital and residential care facilities: a cross-sectional study. BMJ Open 2013;3(8):e002948. | Didn’t develop or validate a prediction model |
| Chen TY, et al. Nutritional Status Predicts Injurious Falls Among Community-Dwelling Older Adults: Does Sex Matter? Journal of Applied Gerontology 2023;42(11):2207-2218. | Didn’t develop or validate a prediction model |
| Ciulla SA. Development of a comprehensive falls prevention model in a long-term care facility. Physical & Occupational Therapy in Geriatrics 2002;21(1):35-49. | Didn’t develop or validate a prediction model |
| Clemson L, et al. Predictors of injurious falls and fear of falling differ: an 11-year longitudinal study of incident events in older people. Journal of Aging & Health 2015;27(2):239-256. | Didn’t develop or validate a prediction model |
| Dalla Via J, et al. Machine-Learning Assessed Abdominal Aortic Calcification is Associated with Long-Term Fall and Fracture Risk in Community-Dwelling Older Australian Women. J Bone Miner Res 2023;38(12):1867-1876. | Didn’t develop or validate a prediction model |
| Dowling L, et al. Dynapenic Abdominal Obesity as a Risk Factor for Falls. The Journal of Frailty & Aging 2023;12(1):37-42. | Didn’t develop or validate a prediction model |
| Ek S, et al. Predictors for functional decline after an injurious fall: a population-based cohort study. Aging Clin Exp Res 2021;33(8):2183-2190. | Didn’t develop or validate a prediction model |
| Evans DD, et al. Addressing Fall Risk from the Emergency Department: What Are We Missing? Advanced Emergency Nursing Journal 2021;43(1):2-9. | Didn’t develop or validate a prediction model |
| Fitzpatrick N, et al. The Syncope-Falls Index: a tool for predicting risk of syncope and complex falls in the older adult based on cumulative health deficits. Qjm 2022;115(6):367-373. | Didn’t develop or validate a prediction model |
| Forslund EB, et al. High incidence of falls and fall-related injuries in wheelchair users with spinal cord injury: A prospective study of risk indicators. Journal of Rehabilitation Medicine 2017;49(2):144-151. | Didn’t develop or validate a prediction model |
| Freiberger E, et al. Short physical performance battery is not associated with falls and injurious falls in older persons: longitudinal data of the SCOPE project. Eur Geriatr Med 2024;15(3):831-842. | Didn’t develop or validate a prediction model |
| Ganz DA, et al. Validation of a Rule-Based ICD-10-CM Algorithm to Detect Fall Injuries in Medicare Data. Journals of Gerontology Series A Biological Sciences & Medical Sciences 2024;79(7):glae096. | Didn’t develop or validate a prediction model |
| Gashaw M, et al. Injury Related to Fall and Its Predictors among Medically Diagnosed Adults with Visual Impairment in Ethiopia: An Observational Cross-Sectional Study. Adv Orthop 2021;2021:6686068. | Didn’t develop or validate a prediction model |
| Jana D, et al. Proportion, Pattern, and Predictors of Falls in Older Adults - A Community-based Observational Study in Rural West Bengal. Journal of Mid life Health 2023;14(2):130-138. | Didn’t develop or validate a prediction model |
| Kenis C, et al. Incidence of falls and fall-related injuries and their predictive factors in frail older persons with cancer: a multicenter study. BMC Geriatrics 2022;22(1):877. | Didn’t develop or validate a prediction model |
| Kim GS, et al. Patterns and predictors of fall injury transitions among Korean older adult fallers: a 2-year longitudinal study. Scientific Reports 2022;12(1):22188. | Didn’t develop or validate a prediction model |
| Koski K, et al. Physiological factors and medications as predictors of injurious falls by elderly people: a prospective population-based study. Age & Ageing 1996;25(1):29-38. | Didn’t develop or validate a prediction model |
| Lo J, et al. Effect of upper and lower extremity control strategies on predicted injury risk during simulated forward falls: A study in healthy young adults. Journal of Biomechanical Engineering-Transactions of the Asme 2008;130(4):041015. | Didn’t develop or validate a prediction model |
| Luukinen H, et al. Factors predicting fractures during falling impacts among home-dwelling older adults. Journal of the American Geriatrics Society 1997;45(11):1302-1309. | Didn’t develop or validate a prediction model |
| Machida Y, et al. Factors predicting oral and maxillofacial fractures after falling and factors predicting the duration of treatment. Dental Traumatology 2023;39(5):418-424. | Didn’t develop or validate a prediction model |
| Majumder S, et al. Effects of trochanteric soft tissue thickness and hip impact velocity on hip fracture in sideways fall through 3D finite element simulations. Journal of Biomechanics 2008;41(13):2834-2842. | Didn’t develop or validate a prediction model |
| Malmivaara A, et al. Risk factors for injurious falls leading to hospitalization or death in a cohort of 19,500 adults. American Journal of Epidemiology 1993;138(6):384-394. | Didn’t develop or validate a prediction model |
| Melzer I, et al. Predicting injury from falls in older adults: Comparison of voluntary step reaction times in injured and noninjured fallers - A prospective study. Journal of the American Geriatrics Society 2009;57(4):743-745. | Didn’t develop or validate a prediction model |
| Min L, et al. Measurement of Fall Injury With Health Care System Data and Assessment of Inclusiveness and Validity of Measurement Models. JAMA Network Open 2019;2(8):e199679. | Didn’t develop or validate a prediction model |
| Mintz J, et al. Identification of Fall-Related Injuries in Nursing Home Residents Using Administrative Claims Data. Journals of Gerontology Series a-Biological Sciences and Medical Sciences 2022;77(7):1421-1429. | Didn’t develop or validate a prediction model |
| Mion LC, et al. Is it possible to identify risks for injurious falls in hospitalized patients? Joint Commission Journal on Quality & Patient Safety 2012;38(9):408-413. | Didn’t develop or validate a prediction model |
| Mojtaba M, et al. Downton Fall Risk Index during hospitalisation is associated with fall-related injuries after discharge: a longitudinal observational study. Journal of Physiotherapy 2018;64(3):172-177. | Didn’t develop or validate a prediction model |
| Mondor L, et al. Weather warnings predict fall-related injuries among older adults. Age & Ageing 2015;44(3):403-408. | Didn’t develop or validate a prediction model |
| Nilsson M, et al. Fall Risk Assessment Predicts Fall-Related Injury, Hip Fracture, and Head Injury in Older Adults. Journal of the American Geriatrics Society 2016;64(11):2242-2250. | Didn’t develop or validate a prediction model |
| Pohl P, et al. Community-dwelling older people with an injurious fall are likely to sustain new injurious falls within 5 years - a prospective long-term follow-up study. Bmc Geriatrics 2014;14:120. | Didn’t develop or validate a prediction model |
| Portegijs E, et al. Asymmetrical lower extremity power deficit as a risk factor for injurious falls in healthy older women. Journal of the American Geriatrics Society 2006;54(3):551-553. | Didn’t develop or validate a prediction model |
| Richardson JK, et al. Hip strength: ankle proprioceptive threshold ratio predicts falls and injury in diabetic neuropathy. Muscle & Nerve 2014;50(3):437-442. | Didn’t develop or validate a prediction model |
| Richardson JK, et al. Complex and Simple Clinical Reaction Times Are Associated with Gait, Balance, and Major Fall Injury in Older Subjects with Diabetic Peripheral Neuropathy. American Journal of Physical Medicine & Rehabilitation 2017;96(1):8-16. | Didn’t develop or validate a prediction model |
| Riska KM, et al. Impact of Hearing Aid Use on Falls and Falls-Related Injury: Results From the Health and Retirement Study. Ear and Hearing 2022;43(2):487-494. | Didn’t develop or validate a prediction model |
| Rivan NFM, et al. Cognitive frailty is a robust predictor of falls, injuries, and disability among community-dwelling older adults. BMC Geriatrics 2021;21(1):593. | Didn’t develop or validate a prediction model |
| Rodriguez-Garcia M, et al. Effect of frailty and sarcopenia on the risk of falls and oste oporotic fractures in anun selected population. Revista de Osteoporosis y Metabolismo Mineral 2020;12(3):81-86. | Didn’t develop or validate a prediction model |
| Ryu E, et al. Individual housing-based socioeconomic status predicts risk of accidental falls among adults. Annals of Epidemiology 2017;27(7):415-420. | Didn’t develop or validate a prediction model |
| Salech F, et al. Osteosarcopenia Predicts Falls, Fractures, and Mortality in Chilean Community-Dwelling Older Adults. Journal of the American Medical Directors Association 2021;22(4):853-858. | Didn’t develop or validate a prediction model |
| Sharma S, et al. Predictors of Falls and Fractures Leading to Hospitalization in People With Dementia: A Representative Cohort Study. Journal of the American Medical Directors Association 2018;19(7):607-612. | Didn’t develop or validate a prediction model |
| Shea CA, et al. Inability to Perform the Repeated Chair Stand Task Predicts Fall-Related Injury in Older Primary Care Patients. American Journal of Physical Medicine & Rehabilitation 2018;97(6):426-432. | Didn’t develop or validate a prediction model |
| Shigematsu R, et al. Motor speed and lower extremity strength as predictors of fall-related bone fractures in elderly individuals. Aging Clinical & Experimental Research 2006;18(4):320-324. | Didn’t develop or validate a prediction model |
| Smith TO, et al. Is there an increased risk of falls and fractures in people with early diagnosed hip and knee osteoarthritis? Data from the Osteoarthritis Initiative. International Journal of Rheumatic Diseases 2018;21(6):1193-1201. | Didn’t develop or validate a prediction model |
| Su Q, et al. An analysis of the associated factors for falls, recurrent falls, and fall-related injuries among the older adults in senior Chinese apartments: A cross-sectional study. Geriatric Nursing 2023;52:127-132. | Didn’t develop or validate a prediction model |
| Tinetti ME, et al. Falls, injuries due to falls, and the risk of admission to a nursing home. New England Journal of Medicine 1997;337(18):1279-1284. | Didn’t develop or validate a prediction model |
| Tromp AM, et al. Predictors for falls and fractures in the longitudinal aging study Amsterdam. J Bone Miner Res 1998;13(12):1932-1939. | Didn’t develop or validate a prediction model |
| Tyndall A, et al. Pragmatic development of an evidence-based intensive care unit-specific falls risk assessment tool: The Tyndall Bailey Falls Risk Assessment Tool. Australian Critical Care 2020;33(1):65-70. | Didn’t develop or validate a prediction model |
| Ullah S, et al. Functional data modelling approach for analysing and predicting trends in incidence rates--an application to falls injury. Osteoporosis International 2010;21(12):2125-2134. | Didn’t develop or validate a prediction model |
| Wagner PP, et al. Bone Microarchitecture Decline and Risk of Fall and Fracture in Men with Poor Physical Performance-The STRAMBO Study. Journal of Clinical Endocrinology and Metabolism 2021;106(12):E5180-E5194. | Didn’t develop or validate a prediction model |
| Wallander M, et al. Type 2 Diabetes and Risk of Hip Fractures and Non-Skeletal Fall Injuries in the Elderly: A Study From the Fractures and Fall Injuries in the Elderly Cohort (FRAILCO). Journal of Bone & Mineral Research 2017;32(3):449-460. | Didn’t develop or validate a prediction model |
| Winger ME, et al. Lower Leg Power and Grip Strength Are Associated With Increased Fall Injury Risk in Older Men: The Osteoporotic Fractures in Men Study. Journals of Gerontology Series a-Biological Sciences and Medical Sciences 2023;78(3):479-485. | Didn’t develop or validate a prediction model |
| Wood JM, et al. Risk of Falls, Injurious Falls, and Other Injuries Resulting from Visual Impairment among Older Adults with Age-Related Macular Degeneration. Investigative Ophthalmology & Visual Science 2011;52(8):5088-5092. | Didn’t develop or validate a prediction model |
| Yau RK, et al. Diabetes and Risk of Hospitalized Fall Injury Among Older Adults. Diabetes Care 2013;36(12):3985-3991. | Didn’t develop or validate a prediction model |
| Zeneli A, et al. Fall predictors in hospitalized patients living with cancer: a case-control study. Supportive Care in Cancer 2022;30(10):7835-7843. | Didn’t develop or validate a prediction model |
| Muir SW, et al. Use of the Berg Balance Scale for predicting multiple falls in community-dwelling elderly people: a prospective study. Physical Therapy 2008;88(4):449-459. | Used a single predictor |
| Toyabe S. World Health Organization fracture risk assessment tool in the assessment of fractures after falls in hospital. BMC Health Services Research 2010;10:106. | Used a single predictor |
| Jørgensen V, et al. Falls and fear of falling predict future falls and related injuries in ambulatory individuals with spinal cord injury: a longitudinal observational study. Journal of Physiotherapy (Elsevier) 2017;63(2):108-113. | Not older adults |
| McCoy TH, Jr., et al. Validation of a risk stratification tool for fall-related injury in a state-wide cohort. BMJ Open 2017;7(2):e012189. | Not older adults |
| Piryonesi SM, et al. Predicting falls and injuries in people with multiple sclerosis using machine learning algorithms. Multiple Sclerosis and Related Disorders 2021;49:102740. | Not older adults |
| Powell-Cope G, et al. Sociotechnical probabilistic risk modeling to predict injurious falls in community living centers. J Rehabil Res Dev 2016;53(6):881-892. | Not older adults |
| Sai A, et al. Quantitative sonographic assessment of quadriceps muscle thickness for fall injury prediction in patients undergoing maintenance hemodialysis: an observational cohort study. BMC Nephrology 2021;22(1):1-9. | Not older adults |
| Schniepp R, et al. Fall prediction in neurological gait disorders: differential contributions from clinical assessment, gait analysis, and daily-life mobility monitoring. Journal of Neurology 2021;268(9):3421-3434. | Not older adults |
| Toyabe S-I. Development of a Risk Assessment Tool to Predict Fall-Related Severe Injuries Occurring in a Hospital. Global Journal of Health Science 2014;6(5):70-80. | Not older adults |
| Wong CK, et al. Risk of fall-related injury in people with lower limb amputations: A prospective cohort study. Journal of Rehabilitation Medicine 2016;48(1):80-85. | Not older adults |
| Yaita S, et al. A Simple and Accurate Model for Predicting Fall Injuries in Hospitalized Patients: Insights from a Retrospective Observational Study in Japan. Medical Science Monitor 2023;29:e941252. | Not older adults |

# Supplementary material table 3. Definition and categories of outcome in the included studies

| **Author (Publication year)** | **Outcome** | **Definition** | **Categories** |
| --- | --- | --- | --- |
| Chan et al. (2023) | FRI | Falls requiring inpatient care (codes W01 to W19 in the ICD-10) | Yes/No |
| Chen et al. (2023) | FRI | Falls requiring medical treatment | Yes/No |
| Davis et al. (2017) | FRI | Falls resulted in a fracture, admission to hospital with an injury, or stitches were required, or there was bruising, sprains, cuts, abrasions, or reduction in physical function for at least three days, or the participant sought medical help | Yes/No (All fallers) |
| Duprey et al. (2022) | FRI | Falls resulted in fractures (excluding fingers and toes), dislocations, concussions, intracranial hemorrhage, and other organ traumas (e.g., pneumothorax) | Yes/No |
| Ek et al. (2019) | First FRI | Falls requiring inpatient or outpatient care | Yes/No |
| Engelbart et al. (2022) | Fall-related cervical spine injuries | Falls resulted in cervical spine injuries | Yes/No (All fallers) |
| Frisendahl et al. (2020) | First FRI | Falls requiring inpatient care | Yes/No |
| Frisendahl et al. (2023) | First FRI | Falls requiring inpatient care and not experiencing FRI within 3 years before the examination date | Yes/No |
| Heo et al. (2023) | FRI | Falls resulted in emergency department visit or admission with primary or first secondary diagnostic code of non-pathological fracture | Yes/No |
| Li et al. (2023) | FRI | Falls requiring medical treatment | Yes/No |
| Shimada et al. (2011) | Fall-related fracture | NR | Yes/No |
| Song et al. (2024) | FRI | NR | Yes/No |
| Speiser et al. (2021) | FRI | Falls resulted in a clinical, non-vertebral fracture or a hospital admission | Yes/No |
| Taseh et al. (2024) | Fall-related hip fracture | NR | Yes/No (All fallers) |
| Zhao et al. (2020) | FRI | Falls resulted in an injury based on the classification criteria of the National Database of Nursing Quality Indicators, which includes five levels of injury (none, minor, moderate, major, and death) | Yes/No |

***Note***: FRI, Fall-related injuries; ICD-10, International Statistical Classification of Diseases and Related Health Problems 10th Revision; NR, not reported.

# Supplementary material table 4. Predictors in the final prediction model of the included studies

| **Author (Publication year)** | **Number of candidate predictors (predictors in the final model)** | **Variables included in the final model** |
| --- | --- | --- |
| Chan et al. (2023) | 33 (15) | Log of daily running duration, Steps per day, Usual walking speed, Sex, Whether the participant lives alone, Diagnoses of dementia, Depression, Central nervous system lesion, Dizziness, Parkinson’s disease, Osteoarthritis, Handgrip strength, Choice reaction time, Presence of abnormal sleeping duration, Alternated sleep phase |
| Chen et al. (2023) | 107 (28) | Marital status, Diabetes, Stroke, Liver disease, Basic activity of daily living, Instrumental activity of daily living, Experience of falling, Experience of hip fracture, Smoking, Sleep duration, Antihypertensive medicine, Dyslipidemia medicine, Digestive medicine, Arthritis medicine, Depressive symptoms, Structure of building, Kitchen, Flush toilets, Internet, House tidiness, House temperature, Income, Dental care, Health satisfaction, Life satisfaction, Lung function, Abdominal obesity, Hand strength |
| Davis et al. (2017) | 27 (8) | Age, Sex, Participation in the Otago exercise programme, Cognition/Processing Speed, Cognition/processing speed, Cognition/working memory, Emotional functioning, Physical functioning & activity, Body composition & fall risk profile |
| Duprey et al. (2022) | 232 (2-year risk full model/6-month risk full model: 70; 2-year risk short tool: 5) | **2-year risk full model/6-month risk full model:** Age group, Sex, Race, Visual impairment, Cognitive Function, Activities of daily living, Orthostatic Hypotension, Diabetes mellitus, Hip fracture, Recent fall, Newly admitted in previous 100 days, Depression, Body Mass Index, Makes self understood, Disorganized thinking, Should mood interview be conducted, Behavioral symptoms put individual at risk, Behavioral symptoms interfere with care, Wandering, Wandering intrudes on privacy, Change in behavior, Transfer - support, Walk in room - self-performance, Walk in corridor - support, Locomotion on unit - self-performance, Locomotion on unit - support, Locomotion off unit - self-performance, Locomotion off unit - support, Dressing - self-performance, Eating - self-performance, Limited ROM - upper extremity, Limited ROM - lower extremity, Cane/crutch, Walker, Wheelchair, Ostomy, Urinary continence, Bowel continence, Cancer, Ulcer, UTI in last 30 days, Arthritis, Other fracture, Aphasia, Cerebral palsy, Malnutrition, Schizophrenia, Eye diseases (Cataracts, glaucoma, macular degeneration), Dyspnea at rest, <6month life expectancy, Mechanically altered diet, Skin tear, Pressure-reducing device for chair, Pressure-reducing device for bed, Application of ointments/skin meds, Days receiving antianxiety treatment in last 7, Days receiving antidepressant treatment in last 7, Days receiving hypnotic treatment in last 7, Days receiving anticoagulant treatment in last 7, Days receiving antibiotic treatment in last 7, Days receiving diuretic treatment in last 7, Received oxygen therapy while admitted, Received most recent influenza vaccine, Days receiving SLP therapy in last 7, Days receiving OT therapy in last 7, Days receiving PT therapy in last 7, Days receiving passive ROM therapy in last 7, Days receiving therapy for eating/swallowing, Decision care planning for falls triggered, Hospitalization in 1 year baseline  **2-year risk short tool:** Activities of daily living, Recent fall, Hospitalized in 1 year baseline, Ability to walk in room, History of fractures other than hip |
| Ek et al. (2019) | 26 (4) | Age, Living alone, Instrumental activity of daily living, Balance (1-leg standing test) |
| Engelbart et al. (2022) | 11 (Model including midline tenderness: 3; Model not including midline tenderness: 2) | **Model including midline tenderness:** signs of trauma, focal neurological deficit, midline spinal tenderness  **Model not including midline tenderness:** signs of trauma, focal neurological deficit |
| Frisendahl et al. (2020) | The FIF screening tool with 4 predictors* | Age, Living alone, Instrumental activity of daily living, Balance (1-leg standing test) |
| Frisendahl et al. (2023) | 4 (4) | Age, Living alone, Instrumental activity of daily living, Balance (self-reported balance problems) |
| Heo et al. (2023) | 187 (26) | Sex, Age group, Insurance status, Number of admission or Emergency Department visit, 7 comorbidities (Prior fall-related injury, Hyperlipidemia, Dorsopathy, Parkinson disease, Menopause, Thyroid disease, Urinary incontinence), 13 medication factors (Number of medication, Number of central nervous system depressant, Loop diuretic, Beta-blocker, ACEi/ARB, Sulfonylurea, Thiazolidinedione, Steroid, Vitamin D, Bisphosphonate, Acetylcholine esterase inhibitor, Hormonal chemotherapy, Hematopoietic drugs), and 2 drug-disease interactions (Heart failure and cilostazol, Fracture and central nervous system depressant) |
| Li et al. (2023) | 11 (11) | Short Physical Performance Battery (high, medium, low), Sex, Age group, Activity of Daily Living, History of fall in the past 2 years, Depression, Stroke, Memory-related disease, Sensory status, Muscle weakness, Cognitive function |
| Shimada et al. (2011) | 13 (13) | The Subjective Risk Rating of Specific Tasks (SRRST) category (no risk, low risk, moderate risk, high risk), Age, Sex, Stroke, Knee osteoarthritis and pain, Dementia, Poor vision, Parkinson disease, Use of psychotropics, Urinary incontinence or frequency, Absence of habitual exercise, Use of slippers or sandals, Use of walking aid |
| Song et al. (2024) | 31 (31) | Demographics (Age, Gender, Race), Diagnosis of comorbidities (Abnormalities of gait and mobility, Monoplegia of lower Extr, Leg_weakness, Hemiplegia, Asymmetry, Multiple sclerosis, Osteoporosis, Osteopenia, Osteoporotic fractures, Orthostatic hypotension, Parkinson’s, Sarcopenia, Stroke, Vestibular sx, Vestibular symptoms Meniere.s.dx, Fall injury, Vision impairment, Total_number_of_comorbidities, ifHas_comorbidities), Fall-risk-related medications (DIAZEPAM, DIPHENHYDRAMINE, LORAZEPAM, MECLIZINE, ZOLPIDEM, ifHas_med, Total_number_of_med), Other (Physical therapy, Hospitalization) |
| Speiser et al. (2021) | 129 (DT: 7; RF: 10) | **DT model:** Age, Cognitive Assessment TASKSWITCH reaction time (no switch), body mass index, HVLT delayed recall, 3MSE 2nd recall score, ONEBACK hits-false alarms, ONEBACK true positive rate  **RF model:** Age, Weight, Cognitive Assessment TASKSWITCH reaction time (switch), Functional activities questionnaire sum, Body mass index, Waist circumference, Chair stands, Digit Symbol Substitution Test score, Cognitive Assessment TASKSWITCH reaction time (no switch), Grip strength |
| Taseh et al. (2024) | 12 (4) | The Fracture Risk Assessment Tool (FRAX) score, Race, Fall Frequency, Housing type and transportation of the Social Vulnerability Index (SVI) |
| Zhao et al. (2020) | 18 (5) | History of fractures, Orthostatic hypotension, Functional status, Sedative‐hypnotics, Level of serum albumin |

***Note***: FIF, first-time injurious fall; FROP-Com, falls risk for older people in the community; AGS, the American Geriatrics Society; BGS, the British Geriatrics Society; AAOS, the American College of Orthopaedic Surgeons; DT, decision tree; RF, random forest. *The study externally validated the existing tool.

# Supplementary material table 5. Frequency of predictors in the included studies and their respective proportions

| **Category** | **Predictor** | **Number of studies (n=16)** | **Proportion (%)** |
| --- | --- | --- | --- |
| **Socio-demographics** | Age | 10 | 66.7 |
|  | Sex | 7 | 46.7 |
|  | Living alone | 4 | 26.7 |
|  | Race | 3 | 20.0 |
|  | Marital status | 1 | 6.7 |
|  | Income | 1 | 6.7 |
|  | Insurance | 1 | 6.7 |
| **Nutritional status** | Body mass index | 2 | 13.3 |
|  | Body weight | 1 | 6.7 |
|  | Abdominal obesity | 2 | 13.3 |
|  | Malnutrition | 1 | 6.7 |
|  | Serum albumin level | 1 | 6.7 |
| **Fall or fall injuries history** | History of fall | 4 | 26.7 |
|  | History of fall injuries | 2 | 13.3 |
| **Fall or fracture risk assessment** | Subjective risk rating of specific tasks | 1 | 6.7 |
|  | Fracture Risk Assessment Tool | 1 | 6.7 |
| **Physical abilities** | Activities in daily living | 4 | 26.7 |
|  | Instrumental activities in daily living | 4 | 26.7 |
|  | Basic activities in daily living | 1 | 6.7 |
|  | Ability to walk in room | 1 | 6.7 |
|  | Limited range of motion | 1 | 6.7 |
|  | Grip strength | 3 | 20.0 |
|  | Muscle weakness | 2 | 13.3 |
|  | Paralysis | 1 | 6.7 |
|  | Walking speed | 1 | 6.7 |
|  | Lung function | 1 | 6.7 |
|  | Dyspnea at rest | 1 | 6.7 |
|  | Elimination problems | 3 | 20.0 |
|  | Foot problems and/or inappropriate footwear | 1 | 6.7 |
|  | Dysphagia | 1 | 6.7 |
| **Balance/Gait** | Balance measures or self-perceived balance problems | 3 | 20.0 |
|  | Balance, gait and/or strength combined | 4 | 26.7 |
|  | Walking aid | 2 | 13.3 |
| **Cognition** | Cognitive function | 2 | 13.3 |
|  | Domain specific tests | 1 | 6.7 |
|  | Choice reaction time | 1 | 6.7 |
|  | Processing speed | 1 | 6.7 |
|  | Working memory | 1 | 6.7 |
| **Health conditions** | Number of diseases | 1 | 6.7 |
|  | Parkinson’s disease | 4 | 26.7 |
|  | Stroke | 4 | 26.7 |
|  | History of fracture | 3 | 20.0 |
|  | Orthostatic hypotension | 3 | 20.0 |
|  | Osteoarthritis | 3 | 20.0 |
|  | Dementia | 2 | 13.3 |
|  | Diabetes | 2 | 13.3 |
|  | History of hip fracture | 2 | 13.3 |
|  | Aphasia | 1 | 6.7 |
|  | Cancer | 1 | 6.7 |
|  | Cerebral palsy | 1 | 6.7 |
|  | Central nervous system lesion | 1 | 6.7 |
|  | Dizziness | 1 | 6.7 |
|  | Dorsopathy | 1 | 6.7 |
|  | Hyperlipidemia | 1 | 6.7 |
|  | Liver disease | 1 | 6.7 |
|  | Menopause | 1 | 6.7 |
|  | Multiple sclerosis | 1 | 6.7 |
|  | Osteopenia | 1 | 6.7 |
|  | Osteoporosis | 1 | 6.7 |
|  | Sarcopenia | 1 | 6.7 |
|  | Thyroid disease | 1 | 6.7 |
|  | Vestibular disorders | 1 | 6.7 |
|  | Visual impairment | 3 | 20.0 |
|  | Sensory loss | 1 | 6.7 |
|  | Eye diseases | 1 | 6.7 |
|  | Skin ulcer | 1 | 6.7 |
|  | Skin tear | 1 | 6.7 |
|  | Urinary tract infection in last 30 days | 1 | 6.7 |
| **Medication** | Number of medications | 2 | 13.3 |
|  | Hypnotics or sedatives | 3 | 20.0 |
|  | Antihypertensives | 2 | 13.3 |
|  | Arthritis drugs | 2 | 13.3 |
|  | Diuretics | 2 | 13.3 |
|  | Antiadrenergic Agents | 1 | 6.7 |
|  | Antibiotics | 1 | 6.7 |
|  | Anticoagulants | 1 | 6.7 |
|  | Antidepressants | 1 | 6.7 |
|  | Anxiolytics | 1 | 6.7 |
|  | Ointments/skin medications | 1 | 6.7 |
|  | Anticancer drugs | 1 | 6.7 |
|  | Anti-Dementia drugs | 1 | 6.7 |
|  | Antidiabetic drugs | 1 | 6.7 |
|  | Anti-dyslipidemia drugs | 1 | 6.7 |
|  | Central nervous system depressant | 1 | 6.7 |
|  | Digestive drugs | 1 | 6.7 |
|  | Hematopoietic drugs | 1 | 6.7 |
|  | Psychotropics | 1 | 6.7 |
| **Drug-disease interactions** | Heart failure and cilostazol | 1 | 6.7 |
|  | Fracture and central nervous system depressant | 1 | 6.7 |
| **Mental health** | Depression or depressive symptoms | 4 | 26.7 |
|  | Sleep quality | 2 | 13.3 |
|  | Behavioral symptom | 1 | 6.7 |
|  | Emotional Functioning | 1 | 6.7 |
|  | Memory problems | 1 | 6.7 |
|  | Schizophrenia | 1 | 6.7 |
| **Lifestyle** | Physical activities | 3 | 20.0 |
|  | Smoking | 1 | 6.7 |
| **Environment** | Pressure-reducing device for chair or bed | 1 | 6.7 |
|  | Structure of building | 1 | 6.7 |
|  | Kitchen | 1 | 6.7 |
|  | Flush toilets | 1 | 6.7 |
|  | Internet | 1 | 6.7 |
|  | House tidiness | 1 | 6.7 |
|  | House temperature | 1 | 6.7 |
|  | Housing type and transportation of the Social Vulnerability Index | 1 | 6.7 |
| **Fall injuries characteristics** | Signs of trauma | 1 | 6.7 |
|  | Focal neurological deficit | 1 | 6.7 |
|  | Midline spinal tenderness | 1 | 6.7 |
| **Others** | Inpatient/outpatient visit | 3 | 20.0 |
|  | Received physical therapy | 2 | 13.3 |
|  | <6month life expectancy | 1 | 6.7 |
|  | Decision care planning for falls triggered | 1 | 6.7 |
|  | Received eating/swallowing therapy | 1 | 6.7 |
|  | Received most recent influenza vaccine | 1 | 6.7 |
|  | Received occupational therapy | 1 | 6.7 |
|  | Received oxygen therapy while admitted | 1 | 6.7 |
|  | Received passive range of motion therapy | 1 | 6.7 |
|  | Received speech-language pathology therapy | 1 | 6.7 |
|  | Body composition & fall Risk profile | 1 | 6.7 |
|  | Dental care | 1 | 6.7 |
|  | Health satisfaction | 1 | 6.7 |
|  | Life satisfaction | 1 | 6.7 |
|  | Participation in the Otago exercise programme | 1 | 6.7 |

***Note***: The frequency of the predictor is measured by the number of studies, and for studies that developed multiple models, the predictor may be included in one or more final models. For statistical purposes, two main processes were carried out. First, the names of the same or similar predictors in different studies were unified and simplified as far as possible, such as “Log of daily running duration”, “Steps per day”, “Walker”, and “Absence of habitual exercise”, all of which are classified into “Physical activity”. Second, multiple predictors with high conceptual similarity in the same study may be grouped into one predictor and therefore calculated only once. For example, the “Log of daily running duration” and “Steps per day” were both from one study (Chan et al., 2023), but were classified as “Physical activities” and counted only once.

# Supplementary material table 6. Overall adherence to the TRIPOD+AI of the included studies

| **Study** | **Adherence Score for TRIPOD+AI Abstracts (%)** | **Adherence Score for TRIPOD+AI (%)** |
| --- | --- | --- |
| Chan et al. (2023) | 53.9 | 71.4 |
| Chen et al. (2023) | 53.9 | 60.5 |
| Davis et al. (2017) | 53.9 | 57.1 |
| Duprey et al. (2022) | 92.3 | 64.3 |
| Ek et al. (2019) | 69.2 | 61.9 |
| Engelbart et al. (2022) | 61.5 | 63.0 |
| Frisendahl et al. (2020) | 76.9 | 47.4 |
| Frisendahl et al. (2023) | 61.5 | 31.3 |
| Heo et al. (2023) | 69.2 | 57.5 |
| Li et al. (2023) | 53.9 | 59.5 |
| Shimada et al. (2011) | 30.8 | 55.8 |
| Song et al. (2024) | 38.5 | 52.4 |
| Speiser et al. (2021) | 46.2 | 45.5 |
| Taseh et al. (2024) | 38.5 | 51.2 |
| Zhao et al. (2020) | 76.9 | 67.4 |


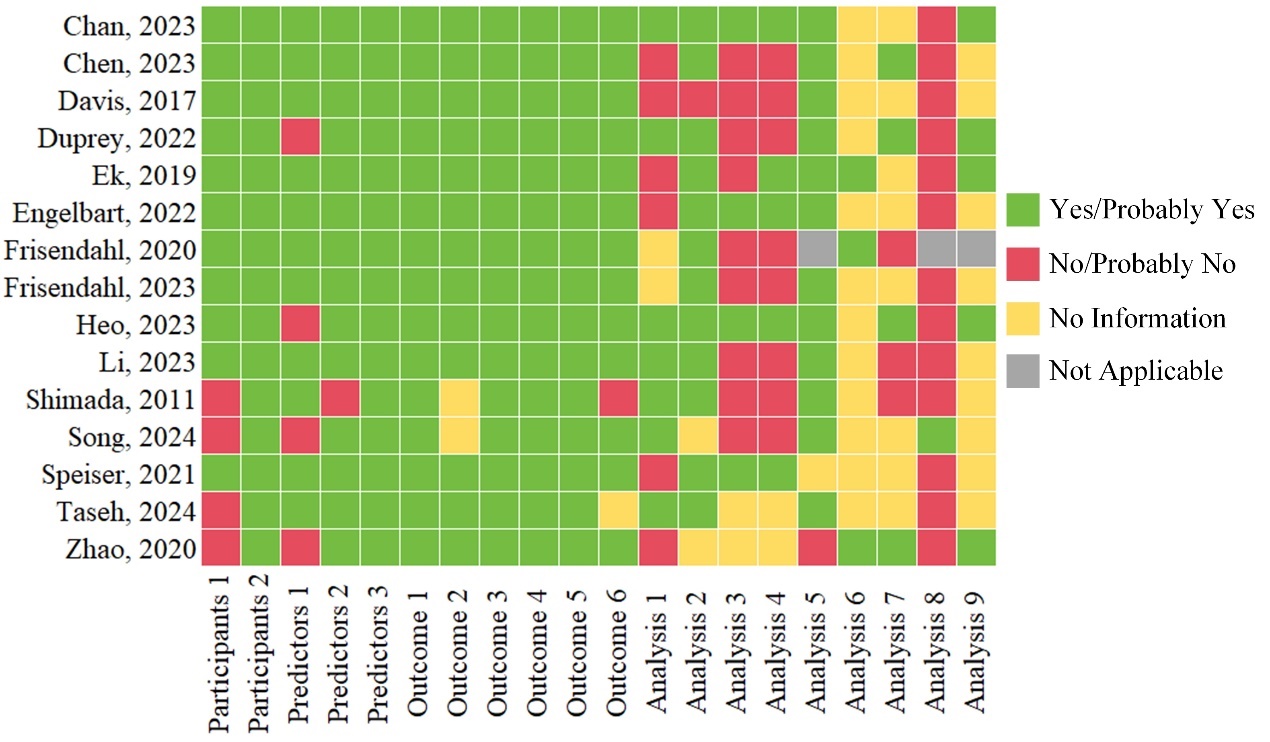


# Supplementary material figure 1. Detailed PROBAST domain assessment results for risk of bias


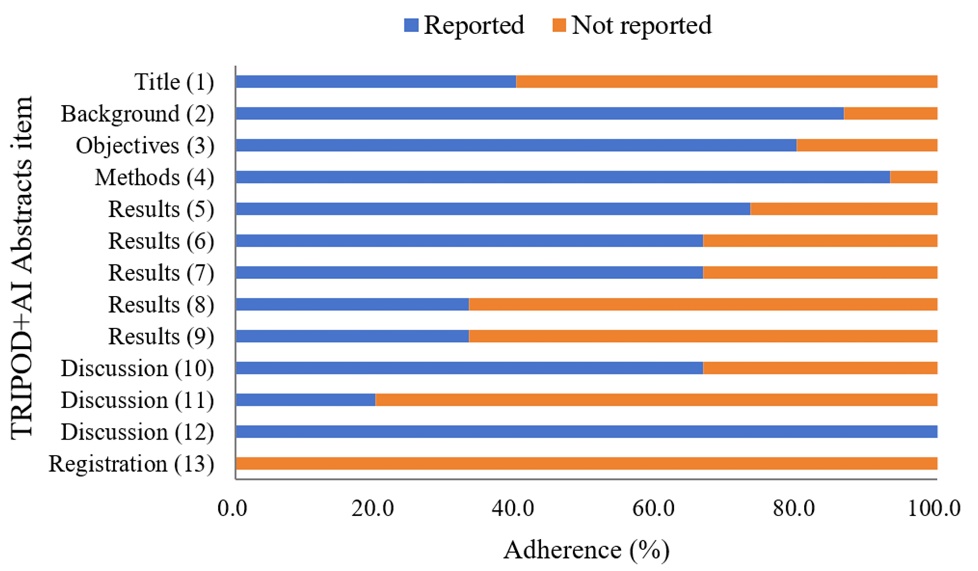


# Supplementary material figure 2. Adherence to the TRIPOD+AI for Abstracts checklist across the items


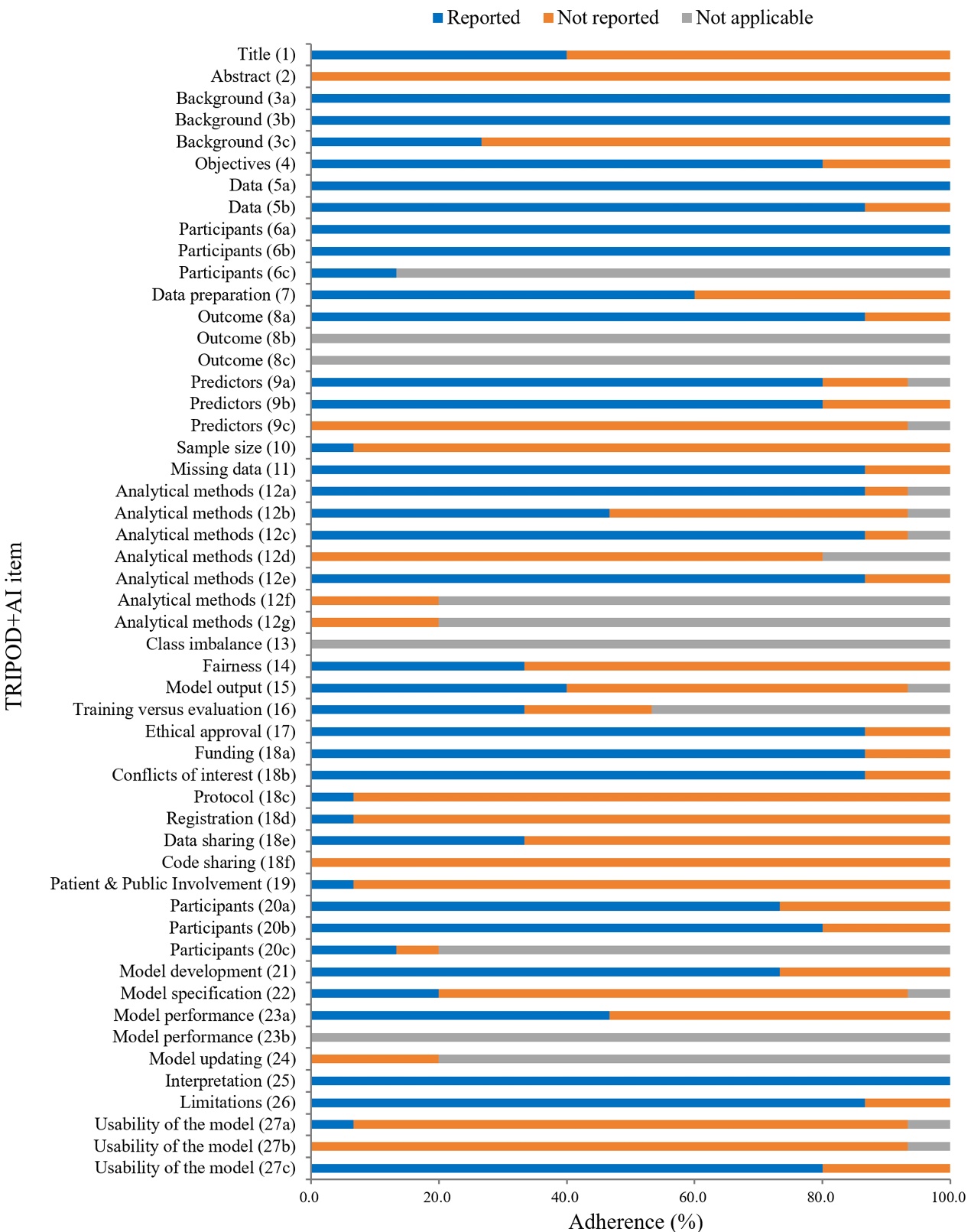


# Supplementary material figure 3. Adherence to the TRIPOD+AI checklist across the items
